# Robust modeling and evidence-based evaluation method for a active distribution network with EVs and CHPs

**DOI:** 10.1038/s41598-025-25084-3

**Published:** 2025-11-21

**Authors:** Kuineng Chen, Jingheng Yuan, Zikang Fang, Yunshou Mao

**Affiliations:** 1 Hunan Engineering Research Center of Special Robot Control Technology and Equipment in Complex Environment, Xiangtan, China; 2 Hunan Vocational Institute of Technology, Xiangtan, China; 3 School of Electronic Information and Electrical Engineering, Huizhou University, Huizhou, China; 4 Huizhou Power Supply Bureau, Guangdong Power Grid Corporation, Huizhou, China

**Keywords:** Active distribution network, Electric vehicles, Robust optimization, Demand response, Status assessment, Electrical and electronic engineering, Renewable energy

## Abstract

Promoting the increase of the proportion of new energy into the power grid and improving the utilization rate of energy has become the urgent needs of the world. To reduce the impact of renewable energy output prediction errors on the distribution network, this paper considers the charging and discharging behaviors of electric vehicles (EVs) and demand responses, and proposes a robust optimization model for the distribution network with a three-stage framework. In the day-ahead stage, a day-ahead optimization operation model is constructed by promoting load shifting through price-based demand response, minimizing daily operation costs while accounting for new energy uncertainty via penalty costs for supply-demand imbalances. In the intraday stage, an intraday rolling optimization model is developed by integrating EV charging/discharging responses, refining the day-ahead plan with higher-resolution forecasts to enhance renewable energy absorption and load balancing. In the real-time stage, a real-time adjustment model utilizes incentive-based demand response to smooth short-term fluctuations in renewable output, ensuring voltage stability and operational flexibility. Experimental simulations on the IEEE 33-bus test system demonstrate that the proposed three-stage cooperative operation strategy significantly promotes renewable energy consumption, reduces operational costs, and improves the reliability of active distribution network (ADN) operation.

**Supplementary Information:**

The online version contains supplementary material available at 10.1038/s41598-025-25084-3.

## Introduction

Recently, there has been a growing recognition of the potential for demand-side resources to contribute to power system dispatch. Incorporating demand response mechanisms into the optimal scheduling of distribution networks not only helps to mitigate the challenges of wind and solar curtailment in the operation of modern power systems, but also facilitates the efficient use of renewable energy and the strategic allocation of resources.

With the large-scale integration of EVs and various distributed energy resources, such as distributed generation, controllable loads, energy storage, and demand-side management, the traditional distribution network has evolved into an active distribution network^1,2^. Through the integration of energy management systems, it is now possible to significantly reduce losses and optimize the resources utilization.

The increasing presence of EVs in the automotive sector is reshaping the future of power systems, presenting both unprecedented opportunities and a series of challenges. The rise in the number of EVs may impose additional burdens on the power grid, particularly during peak charging times, as electricity demand surges. However, the widespread adoption of EVs also offers new flexibility in the power system, such as in demand-side management by adjusting charging times to balance grid loads and serving as distributed energy storage units^3^. Enhancing the energy utilization rate of active distribution networks is achieved by harmonizing the interactions among power generation, demand response, and energy storage systems. Electric vehicles function as both power sources and loads, and additionally, they act as mobile energy storage units. The strategic management of EV charging is crucial for optimizing the energy management within active distribution networks.

### Literature review

The evolution of traditional distribution networks into active distribution networks (ADNs) has been driven by the integration of distributed energy resources (DERs), electric vehicles, and demand response mechanisms. This section synthesizes key research in three interconnected areas—EV integration, renewable energy accommodation, and multi-time-scale optimization—to contextualize the gaps addressed by this study.

#### EV integration and grid impacts

The mass adoption of EVs presents a dual challenge: uncoordinated charging exacerbates grid stress, while coordinated management unlocks flexibility as mobile energy storage. Early studies highlighted risks of unregulated EV integration, such as peak load surges, transformer overloads, and node voltage deviations^4,5^. Solutions initially focused on grid upgrades, such as line capacity expansion^6^, but cost inefficiencies spurred shifts toward demand-side coordination.

Research on EV behavior modeling has advanced from individual charging profiles to aggregated management. Statistical analyses confirm that EV off-grid/on-grid times and driving distances follow normal distributions, enabling clustering techniques to group EVs with similar patterns. Methods like Latin hypercubic sampling and Gaussian distance-based clustering^7,8^ have improved aggregation accuracy, laying the groundwork for vehicle-to-grid (V2G) applications. However, most studies treat EVs as standalone resources, overlooking their synergy with other DERs across time scales—a critical gap in multi-stage optimization.

#### Renewable energy accommodation in ADNs

High-proportion renewable energy introduces volatility that challenges distribution network stability. Key barriers include forecast errors, curtailment losses, and voltage fluctuations^9–11^, driving research on uncertainty mitigation. Power electronic devices (e.g., SVCs) enhance renewable grid-connection stability by regulating reactive power^12^. Moreover, high-resolution forecasting algorithms, including deep learning models such as LSTM (Long Short-Term Memory) networks^13^, Transformer architectures^14^, and hybrid neural networks like Spatial-Temporal Transformers^15^, have significantly reduced prediction errors by capturing both temporal dependencies and spatial correlations in renewable energy output. These advanced algorithms leverage multi-source data to improve accuracy across time scales, from day-ahead to real-time. Meanwhile, robust optimization models, such as two-stage and adaptive robust frameworks, further hedge against residual uncertainties that persist despite improved forecasts, ensuring distribution networks can operate stably under worst-case scenarios of renewable volatility^16^.

Beyond algorithmic and device-based solutions, energy storage systems (ESSs) play a pivotal role in bridging the gap between intermittent renewable generation and variable demand. Stationary ESSs, such as lithium-ion batteries and flywheels, store excess renewable energy during off-peak periods and discharge during high demand, directly reducing curtailment^17^. Notably, electric vehicles—when aggregated as mobile energy storage via V2G technology—extend this capability: their distributed nature allows them to absorb localized renewable surpluses and feed power back to the grid during peak hours, effectively acting as a flexible buffer^18^. This synergy between stationary storage and EVs has emerged as a cost-effective strategy to improve renewable absorption, though most studies focus on electrical storage alone, overlooking the potential of multi-energy systems.

#### Comparative summary of related studies

To better illustrate the research gaps and the contributions of this paper, a comparative summary of existing literature is provided in Table 1.

**Table 1:** Comparative summary of related works.

Study	Focus area	EV modeling	Multi-time scale	Uncertainty handling	Evaluation method	Contribution of this paper
^4,5^	EV grid impact	Individual	No	No	No	Aggregated EV clusters + multi-time scale
^9–11^	Renewable integration	No	No	Probabilistic	Economic only	Robust optimization + tri-time scale
^12^	Voltage stability	No	No	No	No	SVC constraints + voltage stability
^13–15^	Forecasting	No	No	Not considered	No	High-resolution forecasting integrated
^16^	Robust optimization	No	Two-stage	Box uncertainty	Economic	Three-stage + evidence-based evaluation
^17^	ESS and EV	Aggregated	No	Robust	No	Explicit multi-stage V2G scheduling
This paper	ADN with EVs & CHPs	Clustered EVs	Three-stage	Robust + multi-source	Set pair + evidence theory	Comprehensive modeling and evaluation

### Research gaps and paper contributions

With the increasing demand for electric vehicles and distributed generation DG, addressing the optimal dispatch challenges within ADNs is essential to ensure a viable and efficient path forward. However, the literature review has revealed several gaps that warrant attention:

1. Handling the uncertainty: A significant gap in the literature is the effective management of DG-side and load-side uncertainty, particularly with the integration of intermittent renewable energy sources and the variability of load demand. While some studies have proposed models and optimization strategies to address these uncertainties, there is a need for more comprehensive and flexible approaches that can adapt to the complex correlations between sources and loads, especially given the limitations of single-method modeling approaches.
2. Evaluation criteria: Beyond economic metrics, there is a need for a more comprehensive evaluation approach. This paper proposes a set pair analysis and evidence theory-based method for assessing the multi-stage operation of ADNs, offering a broader perspective on network performance.

This paper addresses the aforementioned gaps with the following key contributions:

1. Tri-time scale optimization: We propose a robust tri-time scale optimization model that integrates EV charging/discharging and demand response, enhancing the resilience of ADNs to renewable energy forecast errors.
2. Economic and reliability improvement: Our model, tested on the IEEE 33-bus test system, significantly improves the economic efficiency and operational reliability of ADNs, demonstrating its practical applicability in network management.
3. Evidence-based evaluation: We develop an evaluation model based on set pair analysis and evidence theory, providing a quantifiable status index that validates the superiority of our multi-stage operation approach in terms of economic and stability performance.

## Electric vehicle charging and discharging model

The SOC of a single EV is related to the data of off-grid time (start of driving time), grid-connected time (end of driving time), and traveling distance. EVs have dual characteristics of load and power source. According to statistical studies, the off-grid time, grid-connected time, and driving distance of electric vehicles follow normal distributions, and their probability density functions are as follows, respectively^17^:

1 $${f_{{\text{st}}}}(x)=\left\{ {\begin{array}{*{20}{l}} {\frac{1}{{{\delta _{{\text{st}}}}\sqrt {2\pi } }}{\operatorname{e} ^{ - \frac{{{{(x - {\mu _{{\text{st}}}})}^2}}}{{2\delta _{{{\text{st}}}}^{2}}}}}}&{0<x \leqslant {\mu _{{\text{st}}}}+12} \\ {\frac{1}{{{\delta _{{\text{st}}}}\sqrt {2\pi } }}{\operatorname{e} ^{ - \frac{{{{(x - 24 - {\mu _{{\text{st}}}})}^2}}}{{2\delta _{{{\text{st}}}}^{2}}}}}\;\;}&{\;{\mu _{{\text{st}}}}+12<x \leqslant 24} \end{array}} \right.$$

2 $${f_{{\text{end}}}}(x)=\left\{ {\begin{array}{*{20}{l}} {\frac{1}{{{\delta _{{\text{end}}}}\sqrt {2\pi } }}{\operatorname{e} ^{ - \frac{{{{(x+24 - {\mu _{{\text{end}}}})}^2}}}{{2\delta _{{{\text{end}}}}^{2}}}}}}&{\;\;0<x \leqslant {\mu _{{\text{end}}}} - 12} \\ {\frac{1}{{{\delta _{{\text{end}}}}\sqrt {2\pi } }}{\operatorname{e} ^{ - \frac{{{{(x - {\mu _{{\text{end}}}})}^2}}}{{2\delta _{{{\text{end}}}}^{2}}}}}\;\;}&{\;{\mu _{{\text{end}}}} - 12<x \leqslant 24} \end{array}} \right.$$

3 $${f_{\text{s}}}(x)=\frac{1}{{{\delta _{\text{s}}}x\sqrt {2\pi } }}{\operatorname{e} ^{ - \frac{{{{(\ln x - {\mu _{\text{s}}})}^2}}}{{{\text{2}}\delta _{{\text{s}}}^{2}}}}}$$

where ${f_{{\text{st}}}}(x)$, ${f_{{\text{st}}}}(x)$ and ${f_{{\text{st}}}}(x)$ are the probability density function of EV off-grid time, grid-connected time and distance driven, respectively; $\mu _{{{\text{st}}}}^{{}}$, $\mu _{{{\text{end}}}}^{{}}$ and $\mu _{{\text{d}}}^{{}}$ denote the off-grid time, grid-connected time, and the expected value of the distance driven, respectively; $\delta _{{{\text{st}}}}^{{}}$, $\delta _{{{\text{end}}}}^{{}}$ and $\delta _{{\text{d}}}^{{}}$ denote the standard deviation of off-grid time, on-grid time, and distance driven, respectively.

The state of charge of electric vehicles is related to distance driven:

4 $$SOC=SO{C_0} - \frac{{{f_{\text{s}}}{P_{\text{s}}}}}{{{E_{{\text{EV}}}}}}$$

where $SO{C_0}$ indicates the state of charge at the moment the EV ends charging or discharging. ${P_{\text{s}}}$ and ${E_{{\text{EV}}}}$ denote the energy consumed per kilometer driven by an EV and the capacity of the EV battery, respectively.

Electric vehicles with similar running characteristics are arranged in the same aggregator for unified scheduling management through clustering. In this regard, parameter data (e.g., off-grid time, on-grid time, and end-charge state) of each EV are sampled using the Latin hypercubic sampling method. The Euclidean distance between data points is calculated according to expression (5), and then the Gaussian distance between two points is calculated according to expression (6), which in turn constitutes the similarity matrix ***K***.

5 $${D_{ij}}=\left\| {{x_i} - {x_j}} \right\|$$

6 $${K_{ij}}=\exp ( - \frac{{D_{{ij}}^{2}}}{{2{\varsigma ^2}}})$$

where *D*_*ij*_ represents the Euclidean distances for data of arbitrary EV *i* and *j*; *K*_*ij*_ represents the elements of the similarity matrix.

The Laplacian matrix can be obtained from the similarity matrix and further the regularized Laplacian matrix ***L*** is obtained. Calculate the eigenvectors corresponding to the first *i* largest eigenvalues of the Laplacian matrix ***L***, and use them as the columns to construct the matrix. Convert the row vectors of the matrix into unit vectors to obtain the matrix ***B***. Consider each row of the matrix as a point, and use the k-means algorithm to classify it into *i* class clusters.

Together, Eqs. (5) and (6) enable the aggregation of large-scale EVs into manageable clusters with homogeneous behavior. This reduces computational complexity in the multi-time-scale optimization model, as each cluster can be scheduled as a single entity rather than individual EVs.

After the above steps, different clusters represent different EV charging and discharging behaviors, and the cluster center of each cluster represents the typical characteristics of the EV charging and discharging behaviors of the class.

## A tri-stage optimal scheduling model for an ADN

In order to obtain an intra-day scheduling plan for an ADN with EVs and CHPs under multiple uncertainties, the collaborative scheduling optimization framework proposed in this paper contains both upper- and lower-level optimization models.

### Day-ahead robust optimization model

The ADN operation optimization model takes the minimum daily operation cost as the objective function. The daily operation cost ${f^{{\text{DA}}}}$ includes the cost of purchased electricity $f_{{{\text{grid}}}}^{{{\text{DA}}}}$, purchased natural gas $f_{{{\text{gas}}}}^{{{\text{DA}}}}$, network loss cost, price-based demand response cost^19–21^ and new energy curtailment cost, and the specific expression for the minimum daily operation cost is:

7 $$\hbox{min} {f^{{\text{DA}}}}=f_{{{\text{grid}}}}^{{{\text{DA}}}}+f_{{{\text{gas}}}}^{{{\text{DA}}}}+f_{{{\text{loss}}}}^{{{\text{DA}}}}+f_{{{\text{DR1}}}}^{{{\text{DA}}}}+f_{{{\text{curtail}}}}^{{{\text{DA}}}}{\text{ }}$$

The expression of $f_{{{\text{grid}}}}^{{{\text{DA}}}}$, $f_{{{\text{gas}}}}^{{{\text{DA}}}}$, $f_{{{\text{loss}}}}^{{{\text{DA}}}}$, $f_{{{\text{DR1}}}}^{{{\text{DA}}}}$ can be shown as:

8 $$f_{{{\text{grid}}}}^{{{\text{DA}}}}=\sum\limits_{{t=1}}^{T} {\sum\limits_{{j \in {\Omega ^{{\text{grid}}}}}} {\pi _{t}^{{{\text{grid}}}}} P_{{j,t}}^{{{\text{grid}}}}}$$

9 $$f_{{{\text{gas}}}}^{{{\text{DA}}}}=\sum\limits_{{t=1}}^{T} {\sum\limits_{{j \in {\Omega ^{{\text{CHP}}}}}} {{\pi ^{_{{{\text{gas}}}}}}\left( {F_{{j,t}}^{{{\text{GT}}}}+F_{{j,t}}^{{\text{B}}}} \right)} }$$

10 $$f_{{{\text{loss}}}}^{{{\text{DA}}}}=\sum\limits_{{t=1}}^{T} {\sum\limits_{{ij \in {\Omega ^{{\text{line}}}}}} {{\pi ^{{\text{loss}}}}I_{{ij,t}}^{2}{r_{ij}}} }$$

11 $$f_{{{\text{IDR1}}}}^{{{\text{DA}}}}=\sum\limits_{{t=1}}^{T} {\sum\limits_{{j \in {\Omega ^{{\text{DR}}}}}} {{\pi ^{_{{{\text{IDR1}}}}}}P_{{j,t}}^{{{\text{DR}}1}}} }$$

where ${\Omega ^{{\text{grid}}}}$, ${\Omega ^{{\text{CHP}}}}$, ${\Omega ^{{\text{line}}}}$ and ${\Omega ^{{\text{DR}}}}$ denote the set of electrical energy input nodes, the set of CHP system nodes, the set of line branches, and the set of load nodes involved in demand response, respectively. $\pi _{t}^{{{\text{grid}}}}$, ${\pi ^{_{{{\text{gas}}}}}}$, ${\pi ^{{\text{loss}}}}$ and ${\pi ^{_{{{\text{IDR1}}}}}}$ denote the time-of-use tariff of the grid, the unit price of natural gas purchased, the unit cost of network losses, and the unit cost of load response under price-based demand response, respectively. $P_{{j,t}}^{{{\text{grid}}}}$, $F_{{j,t}}^{{{\text{GT}}}}$, $F_{{j,t}}^{{\text{B}}}$ and $P_{{j,t}}^{{{\text{DR}}1}}$ denote purchased power, gas turbine natural gas consumption power, gas boiler natural gas consumption power and load shifting power in price-based demand response at node *j* at time *t*, respectively.

In the day-ahead optimal scheduling, the existence of uncertainty in wind power and solar power are considered. The actual wind power or solar power will deviate from the planned wind power or solar power to a certain extent, which will not be conducive to the stability of the operation when this deviation exceeds a certain limit, so it is necessary to set the penalty cost for the supply-demand imbalance due to the uncertainty of all new energy sources and to construct a robust optimization model that minimizes the daily operating cost of the day-ahead optimal scheduling model, which is specified by the following expression is:

12 $$\mathop {\hbox{min} }\limits_{{{\psi _1}}} \mathop {\hbox{max} }\limits_{{{\psi _2}}} (f_{{{\text{grid}}}}^{{{\text{DA}}}}+f_{{{\text{gas}}}}^{{{\text{DA}}}}+f_{{{\text{DR1}}}}^{{{\text{DA}}}}+{C_{{\text{pun}}}})$$

13 $${f_{{\text{pun}}}}=c_{t}^{+}\Delta P_{t}^{+}+c_{t}^{ - }\Delta P_{t}^{ - }$$

14 $$\Delta P_{t}^{+}=\sum\limits_{{j \in {\Omega ^{{\text{PV}}}}\& j \in {\Omega ^{{\text{WT}}}}}} {\hbox{max} \left( {P_{{j,t}}^{{{\text{PV}}}}+P_{{j,t}}^{{{\text{WT}}}} - P_{{j,t}}^{{{\text{PV,r}}}} - P_{{j,t}}^{{{\text{WT}},{\text{r}}}},0} \right)}$$

15 $$\Delta P_{t}^{ - }=\sum\limits_{{j \in {\Omega ^{{\text{pv}}}}\& j \in {\Omega ^{{\text{wi}}}}}} {\hbox{max} \left( {P_{{j,t}}^{{{\text{PV,r}}}}+P_{{j,t}}^{{{\text{WT}},{\text{r}}}} - P_{{j,t}}^{{{\text{PV}}}} - P_{{j,t}}^{{{\text{WT}}}},0} \right)}$$

where ${C_{{\text{pun}}}}$ denotes the cost of penalizing the imbalance between supply and demand due to the uncertainty of new energy generation; $\Delta P_{t}^{+}$ and $\Delta P_{t}^{+}$ the overestimated new energy output power and underestimated new energy output power at node *j* at time *t*; $P_{{j,t}}^{{{\text{PV}}}}$ and $P_{{j,t}}^{{{\text{WT}}}}$ denote the predicted value of wind power and PV power at node *j* at time *t*, respectively; $P_{{j,t}}^{{{\text{PV,r}}}}$ and $P_{{j,t}}^{{{\text{WT,r}}}}$ denotes the actual value of wind power and PV power at node *j* at moment *t*, respectively.

Notably, Eq. (7) is the deterministic base model for day-ahead optimization, calculating operational costs under ideal conditions (assuming perfect forecasts). While Eq. (12) extends this to a robust optimization framework by accounting for renewable energy uncertainty. It minimizes the sum of the base cost (from Eq. 7) and the maximum possible penalty cost *f*_pun_ for supply-demand imbalances caused by forecast errors.

The constraints of day-ahead robust optimization model are as follows:

1. Distribution network power flow constraints.
  The distribution network operation constraints can be handled by the second-order cone relaxation technique as follows:
  16 $$\left\{ \begin{gathered} V_{{j,t}}^{ * }=V_{{j,t}}^{{\text{2}}} \hfill \\ I_{{ij,t}}^{ * }=I_{{ij,t}}^{{\text{2}}} \hfill \\ \end{gathered} \right.$$
  17 $$\sum\limits_{{k \in \delta (j)}} {P_{{jk,t}} - } \sum\limits_{{i \in \pi (j)}} {(P_{{ij,t}} - I_{{ij,t}}^{*} r_{{ij}} ) = P_{{j,t}}^{{{\text{grid}}}} + P_{{j,t}}^{{{\text{WT}}}} + P_{{j,t}}^{{{\text{PV}}}} + P_{{j,t}}^{{{\text{GT}}}} + P_{{j,t}}^{{{\text{dis}}}} - P_{{j,t}}^{{{\text{ch}}}} - P_{{j,t}}^{{{\text{load}}}} + P_{{j,t}}^{{{\text{DR1}}}} }$$
  18 $$\sum\limits_{{k \in \delta (j)}} {{Q_{jk,t}}} - \sum\limits_{{i \in \pi (j)}} {({Q_{ij,t}} - I_{{ij,t}}^{ * })=Q_{{j,t}}^{{{\text{grid}}}}+Q_{{j,t}}^{{{\text{SVC}}}} - Q_{{j,t}}^{{{\text{load}}}}}$$
  where $\delta (j)$ and $\pi (j)$ denote the set consisting of the end nodes of the branch with the first end node *j* and the set consisting of the first end nodes of the branch with the end node *j*, respectively. $V_{{j,t}}^{ * }$ and $I_{{ij,t}}^{ * }$ represent the transformed node voltage and branch current, respectively.
2. Distribution network node voltage constraints.
  The balance equation constraints for the voltage drop between nodes are satisfied as shown in Expression (19). After the second-order cone relaxation operation, the following relationships should also be satisfied as shown in Expression (20).
  19 $$V_{{j,t}}^{ * }=V_{{i,t}}^{ * } - 2({P_{ij,t}}{r_{ij}}+{Q_{ij,t}}{x_{ij}})+I_{{ij,t}}^{ * }(r_{{ij}}^{{\text{2}}}+x_{{ij}}^{{\text{2}}})$$
  20 $$\left\| {\begin{array}{*{20}{c}} {{\text{2}}{P_{ij,t}}} \\ {2{Q_{ij,t}}} \\ {I_{{ij,t}}^{ * } - V_{{i,t}}^{ * }} \end{array}} \right\| \leqslant I_{{ij,t}}^{ * }+V_{{i,t}}^{ * }$$
  To ensure the reliability and stability of the distribution network, the voltage fluctuations at each node should be confined within a specific range, and the voltage magnitude at each node must not surpass its permissible upper and lower limits. The voltage constraints can be formulated as follows:
  21 $${(V_{j}^{{{\text{min}}}})^{\text{2}}} \leqslant V_{{j,t}}^{ * } \leqslant {(V_{j}^{{{\text{max}}}})^{\text{2}}}$$
  where $V_{j}^{{{\text{max}}}}$ and $V_{j}^{{{\text{min}}}}$ represent the upper and lower limits of the voltage magnitude at node *j*, respectively.
3. Distribution network branch current constraints.
  To ensure that each branch current does not exceed its prescribed maximum magnitude limit, the following constraints are established:
  22 $$I_{{ij,t}}^{ * } \leqslant {(I_{{ij}}^{{{\text{max}}}})^{\text{2}}}$$
  where $I_{{ij}}^{{{\text{max}}}}$ represents the maximum current magnitude flowing through branch *ij.*
4. SVC output power upper and lower constraints.
  The reactive power output by the SVC must not exceed the upper limit of its capability. The constraints that must be met for the operation of the SVC are:
  23 $${\text{0}} \leqslant Q_{{j,t}}^{{{\text{SVC}}}} \leqslant Q_{j}^{{{\text{SVC,max}}}}$$
  where $Q_{j}^{{{\text{SVC,max}}}}$ denotes the maximum reactive power output of the SVC at node *j*. SVC constraints (23) ensure reactive power support remains within device capabilities, directly mitigating voltage fluctuations caused by intermittent renewable generation and EV charging surges.
5. Other constraints.
  Furthermore, compliance with constraints related to wind and solar power generation, energy storage operations, equipment output limits, thermal load demands, and price-responsive demand response is required. Detailed descriptions of these constraints can be found in references^21–23^; this manuscript does not delve into each constraint individually.

### Intraday optimization model for ADN with EVs

In contrast to the day-ahead stage, the intraday scheduling stage excludes price-responsive demand costs but includes electric vehicle storage charging and discharging costs. The objective function for intraday optimization is as follows:

24 $$\hbox{min} {f^{{\text{ID}}}}=f_{{{\text{grid}}}}^{{{\text{ID}}}}+f_{{{\text{gas}}}}^{{{\text{ID}}}}+f_{{{\text{loss}}}}^{{{\text{ID}}}}+f_{{{\text{V2G}}}}^{{{\text{ID}}}}$$

where $C_{{{\text{V2G}}}}^{{{\text{ID}}}}$ represents the cost of electric vehicle participation in V2G during the intraday stage.

$C_{{{\text{V2G}}}}^{{{\text{ID}}}}$ can be expressed as:

25 $$f_{{{\text{V2G}}}}^{{{\text{ID}}}}=\sum\limits_{{t=1}}^{T} {\sum\limits_{{j \in {\Omega ^{{\text{ev}}}}}} {\pi _{t}^{{{\text{V2G}}}}} } P_{{j,t}}^{{{\text{V2G}}}}$$

where ${\Omega ^{{\text{ev}}}}$ denotes the node where the V2G-enabled electric vehicle cluster is located;$\pi _{t}^{{{\text{V2G}}}}$ represents the unit cost of participating in V2G; $P_{{j,t}}^{{{\text{V2G}}}}$ the power of electric vehicles engaged in V2G at node *j* at time *t* during the intraday stage.

The constraints of intraday optimization model are as follows:

1. Distribution network power flow constraints.
  In the intraday scheduling stage, the distribution network is subject to the following constraints pertaining to active and reactive power distribution:
  26 $$\begin{gathered} \sum\limits_{{k \in \delta (j)}} {{P_{jk,t}} - } \sum\limits_{{i \in \pi (j)}} {({P_{ij,t}} - I_{{ij,t}}^{ * }{r_{ij}})=} P_{{j,t}}^{{{\text{grid}}}}+P_{{j,t}}^{{{\text{WT}}}}+P_{{j,t}}^{{{\text{PV}}}} \hfill \\ \begin{array}{*{20}{c}} {}&{}&{} \end{array}+P_{{j,t}}^{{{\text{gt}}}}+P_{{j,t}}^{{{\text{dis}}}} - P_{{j,t}}^{{{\text{ch}}}} - P_{{j,t}}^{{{\text{load}}}}+P_{{j,t}}^{{{\text{DR1}}}}+P_{{j,t}}^{{{\text{V2G}}}} \hfill \\ \end{gathered}$$
2. EVs constraints.
  To prohibit the simultaneous charging and discharging of electric vehicles, the constraint is mathematically articulated as follows:
  27 $$\gamma _{{j,n,t}}^{{{\text{V2G,dis}}}}+\gamma _{{j,n,t}}^{{{\text{V2G,ch}}}} \leqslant 1$$
  28 $$0 \leqslant P_{{j,n,t}}^{{{\text{V2G,dis}}}} \leqslant \gamma _{{j,n,t}}^{{{\text{V2G,dis}}}}P_{{j,n}}^{{{\text{V2G,max}}}}$$
  29 $$0 \leqslant P_{{j,n,t}}^{{{\text{V2G,ch}}}} \leqslant \gamma _{{j,n,t}}^{{{\text{V2G,ch}}}}P_{{j,n}}^{{{\text{V2G,max}}}}$$
  where $\gamma _{{j,n,t}}^{{{\text{V2G,dis}}}}$ and $\gamma _{{j,n,t}}^{{{\text{V2G,ch}}}}$ are binary variables representing the charging and discharging status of the *n-*th electric vehicle battery at time *t*, respectively.
  The power for charging and discharging of electric vehicle energy storage systems must be bounded by predefined maximum and minimum thresholds. The precise constraints are delineated below:
  30 $$C_{{j,n}}^{{{\text{V2G,min}}}} \leqslant C_{{j,n,t}}^{{{\text{V2G}}}} \leqslant C_{{j,n}}^{{{\text{V2G,max}}}}$$
  31 $$C_{{j,n,t}}^{{{\text{V2G}}}}=C_{{j,n,t - 1}}^{{{\text{V2G}}}}+\frac{{\eta _{{}}^{{{\text{V2G,ch}}}}P_{{j,n,t}}^{{{\text{V2G,ch}}}} - P_{{j,n,t}}^{{{\text{V2G,dis}}}}/\eta _{{}}^{{{\text{V2G,dis}}}} - f_{{j,n,t}}^{{\text{s}}}P_{{j,n}}^{{\text{s}}}}}{{{\text{S}}_{{j{\text{,}}n}}^{{{\text{V2G,max}}}}}}$$

where $C_{{j,n,t}}^{{{\text{V2G}}}}$ denote the state of charge of the *n-*th electric vehicle battery at time *t*; $f_{{j,n,t}}^{{\text{s}}}$ and $P_{{j,n}}^{{\text{s}}}$ denote the travel distance and the power consumption per kilometer of the *n*-th electric vehicle at time *t*, respectively.

Furthermore, in the intraday stage, it is imperative to adhere to the operational constraints established during the day-ahead stage, the specifics of which are not reiterated here for brevity.

### Real-time optimization model for ADN with Incentive-based demand response

After finalizing the intraday V2G plan and keeping energy conversion equipment unchanged, the real-time stage selects loads for incentive-based demand response^22,23^ to smooth out fluctuations in real-time wind and solar power. The real-time optimization objective function is shown below:

32 $$\hbox{min} {f^{{\text{RT}}}}=f_{{{\text{grid}}}}^{{{\text{RT}}}}+f_{{{\text{gas}}}}^{{{\text{RT}}}}+f_{{{\text{loss}}}}^{{{\text{RT}}}}+f_{{{\text{DR2}}}}^{{{\text{RT}}}}$$

where $C_{{{\text{DR2}}}}^{{{\text{RT}}}}$ denotes the cost of load response for incentive-based demand response in the real-time stage.

33 $$f_{{{\text{IDR2}}}}^{{{\text{AT}}}}=\sum\limits_{{t=1}}^{T} {\sum\limits_{{j \in {\Omega ^{{\text{DR}}}}}} {{\pi ^{_{{{\text{IDR2}}}}}}P_{{j,t}}^{{{\text{DR}}2}}} }$$

where ${\pi ^{_{{{\text{IDR2}}}}}}$ represents the unit cost of participating in incentive-based demand response; $P_{{j,t}}^{{{\text{DR}}2}}$ denotes the load response power of incentive-based DR at node *j* at time *t* during the intraday stage.

In the real-time stage, the active and reactive power flow constraints that the distribution network must satisfy are as follows:

34 $$\sum\limits_{{k \in \delta (j)}} {{P_{jk,t}} - } \sum\limits_{{i \in \pi (j)}} {({P_{ij,t}} - I_{{ij,t}}^{ * }{r_{ij}})=} P_{{j,t}}^{{{\text{grid}}}}+P_{{j,t}}^{{{\text{WT}}}}+P_{{j,t}}^{{{\text{PV}}}}+P_{{j,t}}^{{{\text{GT}}}}+P_{{j,t}}^{{{\text{dis}}}} - P_{{j,t}}^{{{\text{ch}}}} - P_{{j,t}}^{{{\text{load}}}}+P_{{j,t}}^{{{\text{DR1}}}}+P_{{j,t}}^{{{\text{V2G}}}}+P_{{j,t}}^{{{\text{DR2}}}}$$

35 $$\sum\limits_{{k \in \delta (j)}} {{Q_{jk,t}}} - \sum\limits_{{i \in \pi (j)}} {({Q_{jk,t}} - I_{{ij,t}}^{ * })=Q_{{j,t}}^{{{\text{grid}}}}+Q_{{j,t}}^{{{\text{SVC}}}} - Q_{{j,t}}^{{{\text{load}}}}}$$

Furthermore, in the real-time stage, it is imperative to adhere to the operational constraints established during the intraday stage, the specifics of which are not reiterated here for brevity.

### Solution methodology

The three stages operate as a sequential adaptive system, where each stage builds on and refines the previous plan using time-scale-specific data, rather than functioning as independent modules.

The core logic is:

1. Day-ahead stage: Develops a baseline plan using 1-hour resolution forecasts, balancing long-term cost efficiency with preliminary uncertainty management.
2. Intraday stage: Adjusts the day-ahead plan using 15-minute resolution forecasts, focusing on medium-term deviations from renewable energy output and EV behavior.
3. Real-time stage: Fine-tunes the intraday plan with 5-minute resolution measurements, eliminating short-term fluctuations to ensure operational stability.

The tri-hierarchical framework propagates decisions across stages as follows:

1. From day-ahead to intraday.

Transferred variables: CHP commitment status, ESS SOC targets, price-DR load-shifting amounts.

(2) From intraday to real-time.

Transferred variables: EV cluster V2G plans, adjusted RES setpoints.

**Fig. 1:**
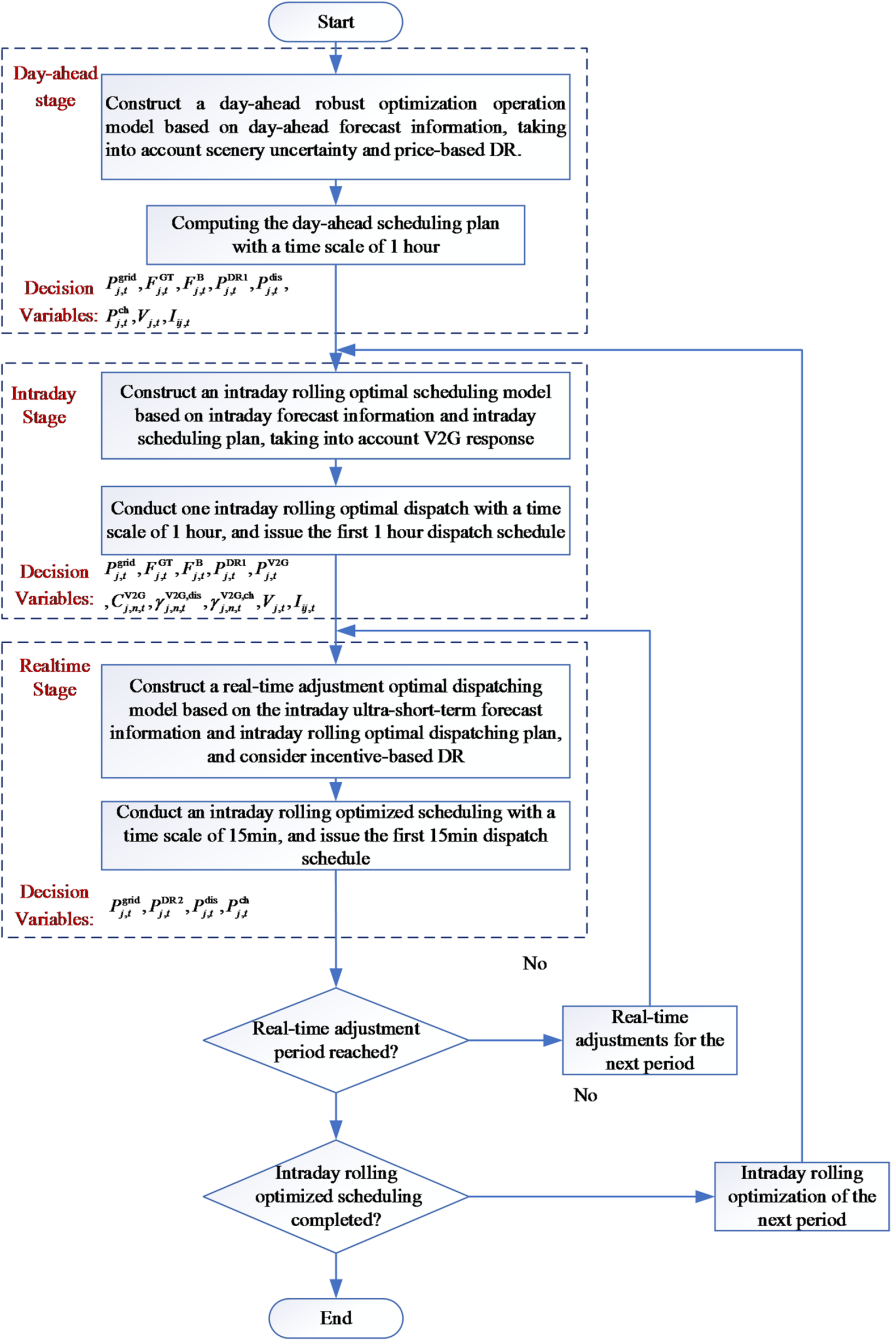
Solution flow chart for multi-stage operation optimization of ADN.

The multi-stage operational optimization of ADN the constructed is divided into three stage: day-ahead optimization, intraday rolling optimization, and real-time adjustment optimization. The specific solution process is depicted in Fig. 1.

1. Solve the master problem
  An auxiliary variable, denoted as *β*, is constructed to decompose the original optimization problem into the master problem as shown in expression (36). After the *i*-th iteration, the solution to the sub-problem, as indicated in expression (42), is incorporated into the objective function of the master problem^24^. The value of *β* should be no less than this objective function value, as shown in expression (37).
  36 $$\mathop {{\text{min}}}\limits_{{{\psi _{\text{1}}}}} {\text{ }}\beta$$
  s.t.
  37 $$\beta \geqslant \sum\limits_{{t=1}}^{T} {(f_{{{\text{grid}}}}^{{{\text{DA}}}}+f_{{{\text{gas}}}}^{{{\text{DA}}}}+f_{{{\text{loss}}}}^{{{\text{DA}}}}+f_{{{\text{DR1}}}}^{{{\text{DA}}}}+f_{{{\text{pun}}}}^{{(i)}})}$$
  where *i* represents the current iteration count of the master problem. $C_{{{\text{pun}}}}^{{(i)}}$ represents denote the penalty cost associated with the imbalance between supply and demand caused by the uncertainty of wind and solar power, as determined after the *i*-th iteration.
  After the *i*-th iteration, the penalty cost returned to the master problem for the imbalance between supply and demand, which includes the costs associated with both overestimating and underestimating the output of wind and solar power, is explicitly formulated as follows:
  38 $$f_{{{\text{pun}}}}^{{(i)}}=c_{t}^{+}\Delta P_{t}^{{(i)+}}+c_{t}^{ - }\Delta P_{t}^{{(i) - }}$$
  39 $$\Delta P_{t}^{{(i)+}}=\sum\limits_{{j \in {\Omega ^{{\text{PV}}}}\& j \in {\Omega ^{{\text{WT}}}}}} {\hbox{max} \{ P_{{j,t}}^{{{\text{PV}}}}+P_{{j,t}}^{{{\text{WT}}}} - P_{{j,t}}^{{{\text{PV,r(}}i{\text{)}}}} - P_{{j,t}}^{{{\text{WT,r(}}i{\text{)}}}},0\} }$$
  40 $$\Delta P_{t}^{{(i) - }}=\sum\limits_{{j \in {\Omega ^{{\text{pv}}}}\& j \in {\Omega ^{{\text{wi}}}}}} {\hbox{max} \{ P_{{j,t}}^{{{\text{PV,r(}}i{\text{)}}}}+P_{{j,t}}^{{{\text{WT,r(}}i{\text{)}}}} - P_{{j,t}}^{{{\text{PV}}}} - P_{{j,t}}^{{{\text{WT}}}},0\} }$$
  where $\Delta P_{t}^{{(i)+}}$ and $\Delta P_{t}^{{(i) - }}$ represent the differences between the scheduled and actual power outputs during the *i*-th iteration, respectively. These differences correspond to the overestimation or underestimation of power, which are critical factors in assessing the penalty costs for supply-demand imbalances.
  The master problem is classified as a Mixed-Integer Linear Programming (MILP) problem. Utilizing commercial solvers within computational frameworks, such as CPLEX, allows for the efficient resolution of MILP problems. After the *k*-th iteration, the optimal solution to the master problem can be obtained as follows:
  41 $$\left\{ {{\beta ^k};P_{j}^{{{\text{grid}},k}},P_{j}^{{{\text{gt}},k}},P_{j}^{{{\text{pv}},k}},P_{j}^{{{\text{wi}},k}},P_{j}^{{{\text{es}},k}},P_{j}^{{{\text{DR1}},k}},Q_{j}^{{{\text{boi}},k}},Q_{j}^{{{\text{hp}},k}},Q_{j}^{{{\text{hes}},k}}} \right\}$$
2. Solve the sub-problem.
  Upon obtaining the solution to the master problem, we utilize this outcome as input for the sub-problem. The subsequent step involves identifying the actual power generation from photovoltaic and wind energy devices. This determination is crucial for maximizing the penalty costs that arise from supply-demand imbalances due to the volatility of wind and solar power output.
  42 $$\mathop {\hbox{max} }\limits_{{{\psi _2}}} \sum\limits_{{t=1}}^{T} {\left\{ {f_{{{\text{grid}}}}^{{{\text{DA}},k}}+f_{{{\text{gas}}}}^{{{\text{DA}},k}}+f_{{{\text{loss}}}}^{{{\text{DA}},k}}+f_{{{\text{aban}}}}^{{{\text{DA}},k}}+f_{{{\text{DR1}}}}^{{{\text{DA}},k}}+c_{t}^{+}\Delta P_{t}^{+}+c_{t}^{ - }\Delta P_{t}^{ - }} \right\}}$$
  s.t.
  43 $$\Delta P_{t}^{+}=\sum\limits_{{j \in {\Omega ^{{\text{pv}}}}{\text{\& }}j \in {\Omega ^{{\text{wi}}}}}} {\hbox{max} \{ P_{{j,t}}^{{{\text{pv}},k}}+P_{{j,t}}^{{{\text{wi}},k}} - P_{{j,t}}^{{{\text{pv}},{\text{r}}}} - P_{{j,t}}^{{{\text{wi}},{\text{r}}}},0\} }$$
  44 $$\Delta P_{t}^{ - }=\sum\limits_{{j \in {\Omega ^{{\text{pv}}}}{\text{\& }}j \in {\Omega ^{{\text{wi}}}}}} {\hbox{max} \{ P_{{j,t}}^{{{\text{pv}},{\text{r}}}}+P_{{j,t}}^{{{\text{wi}},{\text{r}}}} - P_{{j,t}}^{{{\text{pv}},k}} - P_{{j,t}}^{{{\text{wi}},k}},0\} }$$
  45 $$0.9 \times P_{{j,t}}^{{{\text{wi,e}}}} \leqslant P_{{j,t}}^{{{\text{wi,r}}}} \leqslant 1.1 \times P_{{j,t}}^{{{\text{wi,e}}}}$$
  46 $$0.9 \times P_{{j,t}}^{{{\text{pv,e}}}} \leqslant P_{{j,t}}^{{{\text{pv,r}}}} \leqslant 1.1 \times P_{{j,t}}^{{{\text{pv,e}}}}$$
  47 $$\sum\limits_{{t=1}}^{T} {\left| {\frac{{P_{t}^{{{\text{WT,r}}}} - P_{t}^{{{\text{WT,e}}}}}}{{P_{t}^{{{\text{WT,max}}}} - P_{t}^{{{\text{WT,e}}}}}}} \right|} \leqslant {\Gamma _{{\text{WT}}}}$$
  48 $$\sum\limits_{{t=1}}^{T} {\left| {\frac{{P_{t}^{{{\text{PV,r}}}} - P_{t}^{{{\text{PV,e}}}}}}{{P_{t}^{{{\text{PV,max}}}} - P_{t}^{{{\text{PV,e}}}}}}} \right|} \leqslant {\Gamma _{{\text{PV}}}}$$
  After the *k-*th iteration, the optimal solution for the subproblem is obtained as follows:
  49 $$\left\{ {P_{j}^{{{\text{wi,r(k}}+{\text{1)}}}},P_{j}^{{{\text{pv,r(k}}+{\text{1)}}}}} \right\}$$
  With a predefined convergence criterion, the master and subproblems are iteratively solved. The process converges when the objective function value difference between the subproblem and the master problem falls below this threshold, the optimal solution of the original problem are as:
  50 $$\left\{ {{\beta ^k};P_{j}^{{{\text{grid}},k}},P_{j}^{{{\text{gt}},k}},P_{j}^{{{\text{pv}},k+1}},P_{j}^{{{\text{wi}},k+1}},P_{j}^{{{\text{es}},k}},\Delta P_{j}^{{{\text{DR1}},k}},Q_{j}^{{{\text{boi}},k}},Q_{j}^{{{\text{hp}},k}},Q_{j}^{{{\text{hes}},k}}} \right\}$$

## State evaluation methods

When studying multi-stage operational optimization for ADNs, it’s crucial to balance the enhancement of economic efficiency with an in-depth analysis of stability-affecting factors like voltage fluctuation and load peak-to-valley ratios. To precisely evaluate and compare the merits of different strategies on varying time scales, we must convert indicators of economic and stability performance into concrete, quantifiable, and comparable metrics. This approach is essential for validating strategy effectiveness, conducting holistic assessments, and substantiating the viability of the multi-stage optimization strategies discussed in Sect. “A tri-stage optimal scheduling model for an ADN”.

### Determination of evaluation indicators

**Fig. 2:**
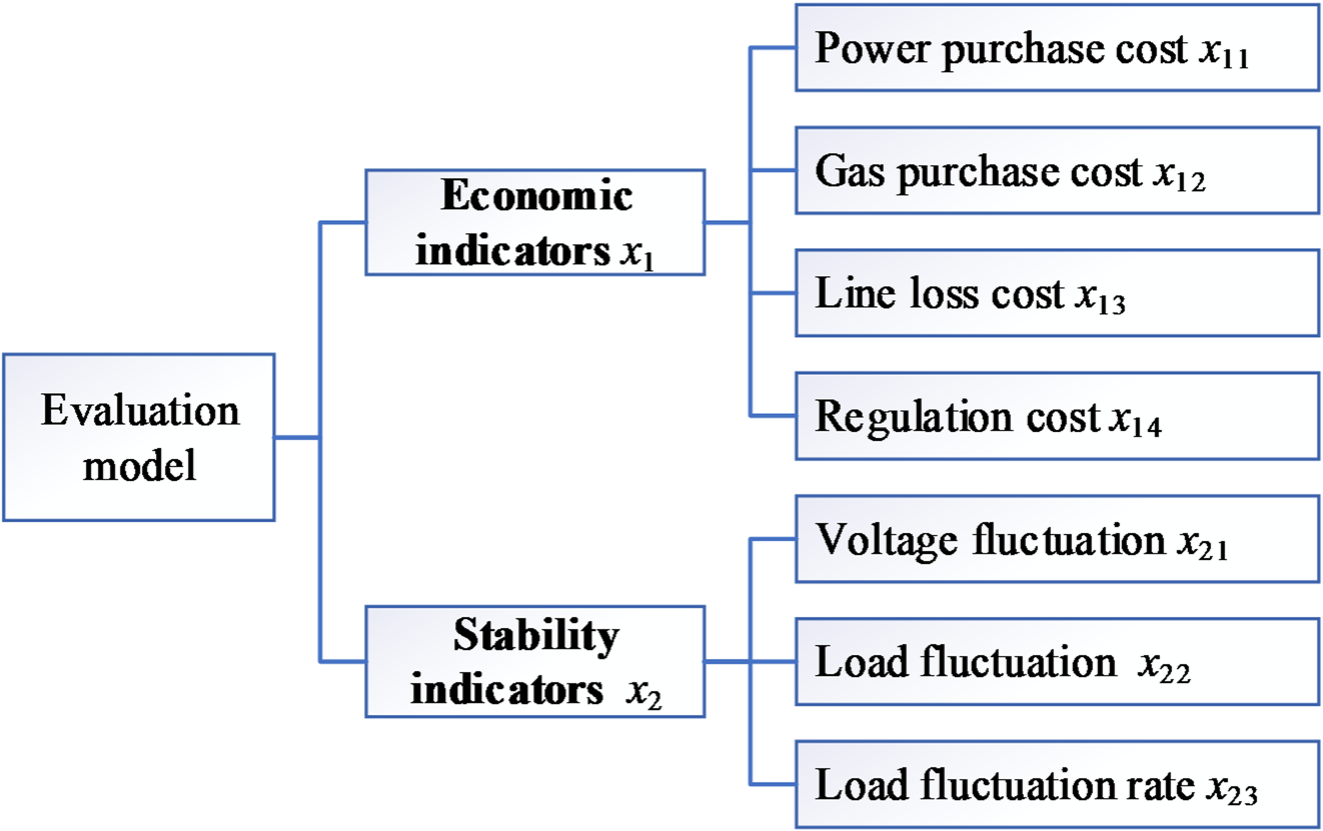
Evaluation model the multi-stage operating status.

To construct a robust index system for evaluating multi-stage operational status, it is imperative to reflect a broad spectrum of benefits, such as economic gains and system security. The chosen indicators must be based on consistent standards and quantifiable methods to ensure their comparability. The model selects indicators comprising 2 primary and 7 secondary metrics, categorized under economic and stability aspects, as shown in Fig. 2. Economic indicators include the costs of electricity and natural gas procurement, network losses, and control unit expenses, while stability indicators cover load and voltage fluctuation amplitudes, as well as load fluctuation rates.

The presence of indicators with varying dimensions in the evaluation model complicates direct comparison and holistic assessment. To resolve this, indicators must be quantified through standardization or normalization, transforming them into dimensionless forms for comparable evaluation:

51 $${Y_{ij}}=\frac{{{X_{ij}} - {X_{i,{\text{min}}}}}}{{{X_{i,\hbox{max} }} - {X_{i,\hbox{min} }}}},{X_{i,\hbox{min} }} \leqslant {X_{ij}} \leqslant {X_{i,\hbox{max} }}$$

52 $${Y_{ij}}=\frac{{{X_{ij}} - {X_{i,{\text{min}}}}}}{{{X_{i,\hbox{min} }} - {X_{i,\hbox{max} }}}},{X_{i,\hbox{min} }} \leqslant {X_{ij}} \leqslant {X_{i,\hbox{max} }}$$

Equation (49) represents the quantification of reverse evaluation indicators, where a smaller value indicates better performance in the corresponding aspect. Equation (50) represents the quantification of positive evaluation indicators, where a larger value indicates better performance in the corresponding aspect. The standardized value of the indicator data is {*Y*_1_, *Y*_2_,…,*Y*_N_}.

The weight of evaluation indicators for the multi-stage operation status of the ADN is obtained using the entropy method. The entropy method is an objective weighting technique based on the principle of information entropy. The specific calculation is completed in the following three steps:

1. Normalization of data: As shown in Expressions (49) and (50) above-mentioned.
2. Calculation of entropy Values: Compute the entropy values for each indicator to measure the degree of uncertainty or information contained within the data:
  53 $${p_{ij}}=\frac{{{Y_{ij}}}}{{\sum\limits_{{i=1}}^{n} {{Y_{ij}}} }}$$
  54 $${E_j}= - \ln {(n)^{ - 1}}\sum\limits_{{i=1}}^{n} {{p_{ij}}\ln {p_{ij}}}$$
  where ${p_{ij}}$ and ${E_j}$ denote intermediate parameters in the calculation of entropy values and the information entropy of each indicator, respectively.
3. Determination of weights: Determine the weights of each indicator based on the calculated entropy values:
  55 $${w_k}=\frac{{1 - {E_k}}}{{n - \sum\limits_{{k=1}}^{n} {{E_k}} }}$$
  where ${w_k}$ denotes the information entropy of each indicator.

### Set pair analysis of index evaluation

Set Pair Analysis is an innovative method for systematic analysis that utilizes the concept of relational degrees to manage uncertainties. It combines qualitative and quantitative elements of decision-making to address problems arising from randomness, ambiguity, and incompleteness, revealing inherent knowledge and patterns^18^. The general process of SPA includes the following steps:

1. Calculate the sameness, opposition, and difference degrees of the set pair.
  56 $$L=\{ A,B\}$$
  57 $$N=S+P+F$$
  58 $$a=\frac{S}{N},b=\frac{F}{N},c=\frac{P}{N}$$
  where *a*, *b*, and *c* denote the identity degree, opposition degree, and difference degree of the set pair, respectively.
2. Calculate the connection degree of the set pair.
  In a specific evaluation system for a particular issue, it is assumed that a characteristic quantity contains *N*_*k*_ indicators and has *K* status levels. The set of characteristic indicators is denoted as *X*, and the set of status levels is denoted as Z. By forming a set pair **L** = {**X**_k_,**Z**}, the connection degree $\mu$ of this set pair is as shown in expression (59).
  59 $$\mu ==a+bi+cj$$
  where *a*_k_, *b*_k_, and *c*_k_ represent the identity degree, opposition degree, and discrepancy degree components, respectively, between the indicators and a specific status level *z*_k_ within the status level set. The discrepancy degree component refers to the degree of difference for the non-identical elements.
  The evaluation system employs fuzzy logic rules to ascertain the degree of association, known as the elemental connection degree, between the quantified outcomes of evaluation indicators and the elemental status levels *K*.
  60 $$\mu =\sum\limits_{{k=1}}^{{{N_k}}} {{w_k}} {\mu _k}$$
  61 $$\mu _{{kl}} = \left\{ \begin{gathered} {\text{1}} + {\text{0}} \cdot i_{1} + 0 \cdot i_{2} + \cdot \cdot \cdot + 0 \cdot i_{{K - 2}} + 0 \cdot j,\;x_{{kl}} \le r_{1} ; \hfill \\ \frac{{r_{1} + r_{2} - 2 \cdot x_{{kl}} }}{{r_{2} - r_{1} }} + \frac{{2 \cdot x_{{kl}} - 2 \cdot r_{1} }}{{r_{2} - r_{1} }} + 0 \cdot i_{2} {\kern 1pt} + \cdot \cdot \cdot + 0 \cdot i_{{K - 2}} + 0j,\;r_{1} \le x_{{kl}} \le \frac{{r_{1} + r_{2} }}{2}; \hfill \\ 0 + \frac{{r_{2} + r_{3} - 2 \cdot x_{{kl}} }}{{r_{3} - r_{1} }}i_{1} {\kern 1pt} + \frac{{2 \cdot x_{{kl}} - r_{1} - r_{2} }}{{r_{3} - r_{1} }}i_{2} + \cdot \cdot \cdot + 0 \cdot i_{{K - 2}} + 0 \cdot j,\;\frac{{r_{1} + r_{2} }}{2} \le x_{{kl}} \le \frac{{r_{2} + r_{3} }}{2}; \hfill \\ \cdot \cdot \cdot \hfill \\ {\text{1}} + {\text{0}} \cdot i_{1} + 0 \cdot i_{2} + \cdot \cdot \cdot + \frac{{2 \cdot r_{{K - 1}} - 2 \cdot x_{{kl}} }}{{r_{{K - 1}} - r_{{K - 2}} }} + \frac{{2 \cdot x_{{kl}} - r_{{K - 2}} - r_{{K - 1}} }}{{r_{{K - 1}} - r_{{K - 2}} }}j,{\kern 1pt} \;\frac{{r_{{K - 2}} + r_{{K - 1}} }}{2} \le x_{{kl}} \le r_{{K - 1}} ; \hfill \\ {\kern 1pt} {\kern 1pt} 0{\kern 1pt} + {\text{0}} \cdot i_{1} + 0 \cdot i_{2} + \cdot \cdot \cdot + 0 \cdot i_{{K - 2}} + 1 \cdot j,\;x_{{kl}} > r_{{K - 1}} {\kern 1pt} ; \hfill \\ \end{gathered} \right.$$

where ${w_k}$ epresents the weight of the indicator. *r*_1_, *r*_2_,…,*r*_k_ denote the boundary values established for the status levels within the evaluation framework.

This paper categorizes the multi-stage ADN operational status into five levels, as shown in Table 2.

**Table 2:** Simulation parameter table.

Status level	Z_1_	…	Z_k−1_	Z_k_
Range of values	(0,*r*_1_)	…	[*r*_*k−2*_,*r*_*k−1*_)	[*r*_*k−1*_,1)

### Index fusion using evidence theory

The Dempster-Shafer (DS) Theory of Evidence, also known as Evidence Theory, is a mathematical framework for dealing with uncertainty^25,26^. This approach exhibits significant capability in handling uncertain information characterized by randomness, fuzziness, inaccuracy, and inconsistency.

Constructing an index fusion model using Evidence Theory involves the following steps:

1. Define the identification framework.
  In this section, the evaluation levels of the ADN’s multi-stage operation status and the uncertainty $\theta$ are defined as the frame of discernment $\Theta$, as shown in expression (62).
  62 $$\Theta ={\text{\{ }}{z_1},{z_2},{z_3},{z_4},{z_5}{\text{,}}\theta {\text{\} }}$$
2. Determine basic belief assignments.
  The function used to calculate the connection degree between the evidence of characteristic quantities and the status levels is the Basic Belief Assignment (BBA) function. The specific expressions are as shown in expressions (63)–(65):
  63 $$m(\emptyset )=0$$
  64 $$0 \leqslant m(A) \leqslant 1$$
  65 $$\sum\limits_{{A \subseteq \Theta }} {m(A)=1}$$
  where *G* represents any subset of the discernment frame, and *m*(*G*) is BBA function.
3. Determine the weight
  The feasibility coefficient is introduced to represent the viability of evidence, with higher coefficients indicating greater feasibility of the corresponding evidence, and vice versa. The definition of the feasibility coefficient and the modification of the BBA function are as follows:
  66 $${\lambda _k}=\lambda \cdot \frac{{{\omega _k}}}{{{\omega _{\hbox{max} }}}}$$
  67 $${\omega _{{\text{max}}}}=\hbox{max} \{ {\omega _1},{\omega _2} \cdot \cdot \cdot {\omega _k} \cdot \cdot \cdot {\omega _M}\}$$
  68 $$\left\{ \begin{gathered} {m_k}(Z){\kern 1pt} {\kern 1pt} {\kern 1pt} ={\lambda _k}{\mu _k}{\kern 1pt} \hfill \\ {m_k}(\theta )=1 - {\lambda _k}{\kern 1pt} {\kern 1pt} \hfill \\ \end{gathered} \right.$$
  where ${\lambda _k}$ represents the viability of evidence for each subset, while $\lambda$ represents the optimal feasibility coefficient. ${\omega _{{\text{max}}}}$ represents the maximum characteristic quantity weight.
4. Evidence convergence and outcome assessment:
  To address the errors stemming from evidential conflicts, this study utilizes an open evidence fusion strategy. Following the acquisition of the fused BBA values, the fusion results are assessed using the principles of plausibility and maximum membership degree. The normalization constant *K* can be calculated as:
  69 $$K=\sum\limits_{{B \cap C \ne \emptyset }} {{m_{\text{1}}}(B){m_2}(C)}$$
  70 $$m(\lambda )=\sum\limits_{{B \cap C=\lambda }} {\frac{{{m_1}(B){m_2}(C)}}{{1 - K}}}$$
  where $m(\lambda )$ is are the basic belief assignments from the first and second pieces of evidence, respectively.

### Overall process of state evaluation

To obtain a more favorable evaluative judgment, this paper equates the membership degrees corresponding to different status levels with numerical scores, as demonstrated in expression (71).

71 $$F={m_r}({L_1}) \times 0+{m_r}({L_2}) \times 30+{m_r}({L_3}) \times 40+{m_r}({L_4}) \times 50+{m_r}({L_5}) \times 100+{m_r}({L_\Theta }) \times 50$$

where ${m_r}({L_i})$ and ${m_r}({L_\Theta })$ represent the membership degrees for different status levels and the membership degrees for uncertainty, respectively. *F* represents the evaluation index, with a range of [0,100].

**Fig. 3:**
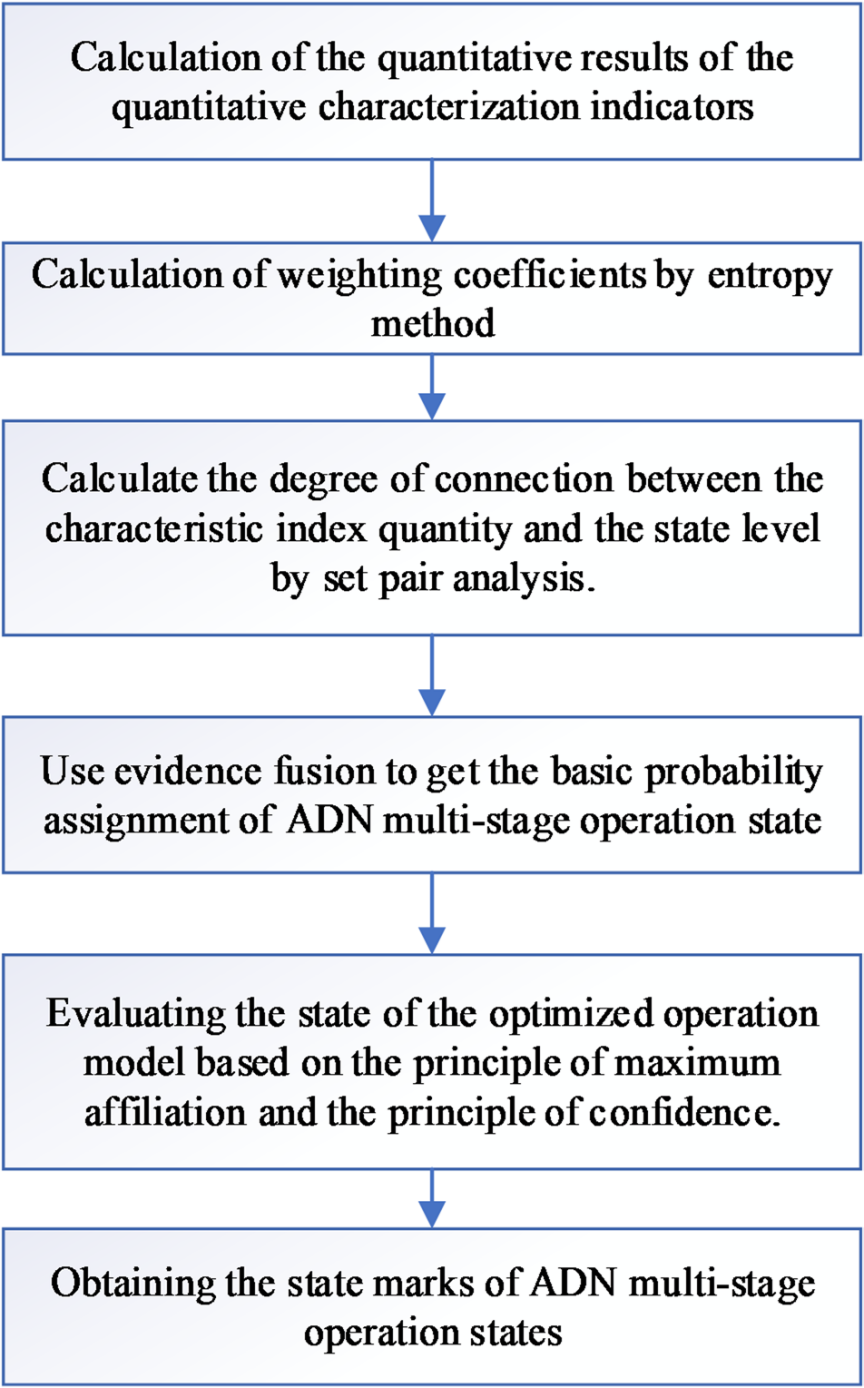
The overall process of evaluating the multi-stage operating status of ADN.

The evaluation process, as illustrated in Fig. 3, involves quantification of characteristic indicators for the ADN multi-stage operation status, determination of weight coefficients using the entropy method, establishment of correlations between indicator quantities and status levels via set pair analysis, derivation of basic probability distributions through evidence fusion and feasibility coefficients, and ultimately, assessment of the ADN’s multi-stage operation status based on the principle of maximum membership degree and credibility. The outcomes are then translated into equivalent scores for a more straightforward evaluation.

## Case study

### Basic settings

**Fig. 4:**
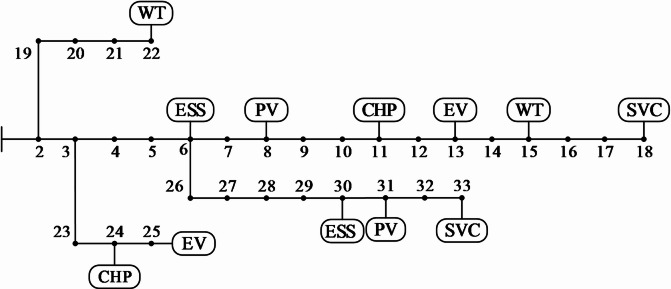
I IEEE test 33-bus distribution system.

The proposed ADN multi-stage optimization model was simulated on the IEEE 33-bus distribution system. Two wind turbine farms were selected to connect at nodes 15 and 22, two photovoltaic farms at nodes 8 and 31, two ESSs at nodes 6 and 30, two CHP systems at nodes 11 and 24, two EV charging stations at nodes 13 and 25, and two SVC systems at nodes 18 and 33. The topology is shown in Fig. 4, and the parameter details of the equipment within the CHP systems are presented in Table A1 in Appendix A (Supplementary Material). The price of natural gas is taken as 2.07 CYN/m^3^, and the calorific value of natural gas is taken as 9.73 kWh/m^3^. The overestimation of energy supply penalty price is 1.5 times the electricity price, and the underestimation of energy supply penalty is 0.5 times the electricity price. The simulation takes 24 h as a scheduling period, with the unit scheduling time being 1 h.

Selected 200 electric vehicles to participate in V2G response, assuming the electric vehicles are of the same model, the battery energy storage charging and discharging power is 3.6 kW, the battery energy storage capacity is 35 kWh, the battery energy storage charging and discharging efficiency is 90%, the depth of discharge is 0.08, the battery SOC is uniformly set to 0.9 when disconnected from the grid, and the power consumption per kilometer of driving distance is 0.25 kW. The output forecast results of wind and photovoltaic renewable energy units at various time scales are shown in Fig. A2 in Appendix (Supplementary Material). The forecasted electrical load output and thermal load output are shown in Figure A2 in Appendix.

### Day-ahead stage optimization result analysis

**Fig. 5:**
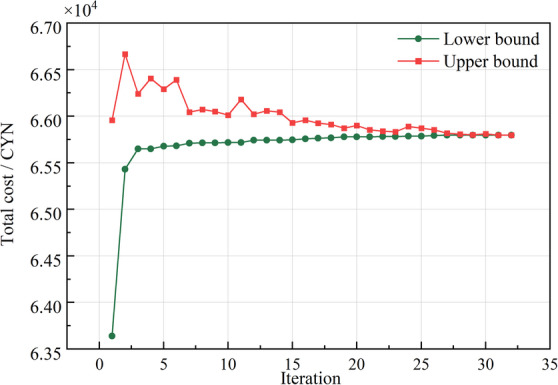
Iterative convergence results of the C&CG algorithm in the day-ahead stage.

In the day-ahead optimization, the Column-and-Constraint Generation method addresses wind and solar uncertainty with a two-stage robust approach. The upper bound reflects the most adverse intraday scenario, and the lower bound aligns with the day-ahead plan. Iterative refinement of the day-ahead plan minimizes operational costs under unfavorable conditions, enhancing robustness. After 32 iterations, the optimal solution reveals a day-ahead total cost of 65,796 CYN, as shown in Fig. 5.

**Fig. 6:**
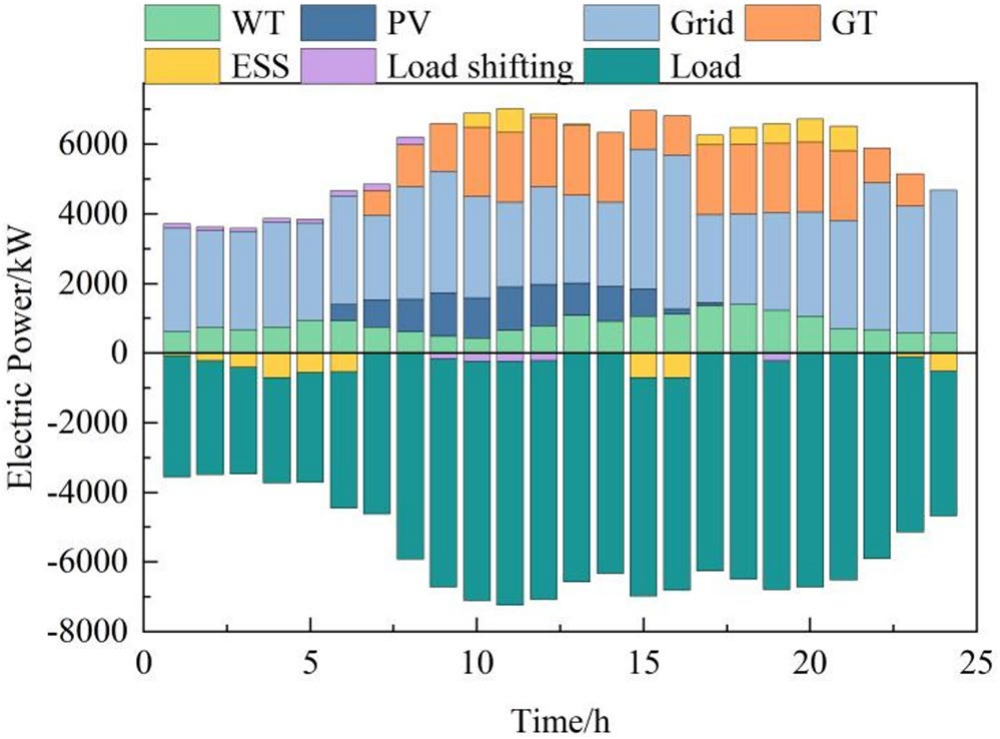
Electric power optimization operation results in the day-ahead stage.

During off-peak tariffs, renewable energy output is low, making grid-purchased electricity cheaper than gas turbine generation. Microgrids thus prioritize grid power and use storage to absorb excess renewables, reducing curtailment. In standard periods, gas turbines and grid purchases jointly meet demand. Peak periods see gas turbines at full output, with demand response and storage discharges to shift loads and alleviate grid stress, minimizing costs and exploiting peak-off-peak price differentials. Day-ahead power optimization outcomes are illustrated in Fig. 6.

**Fig. 7:**
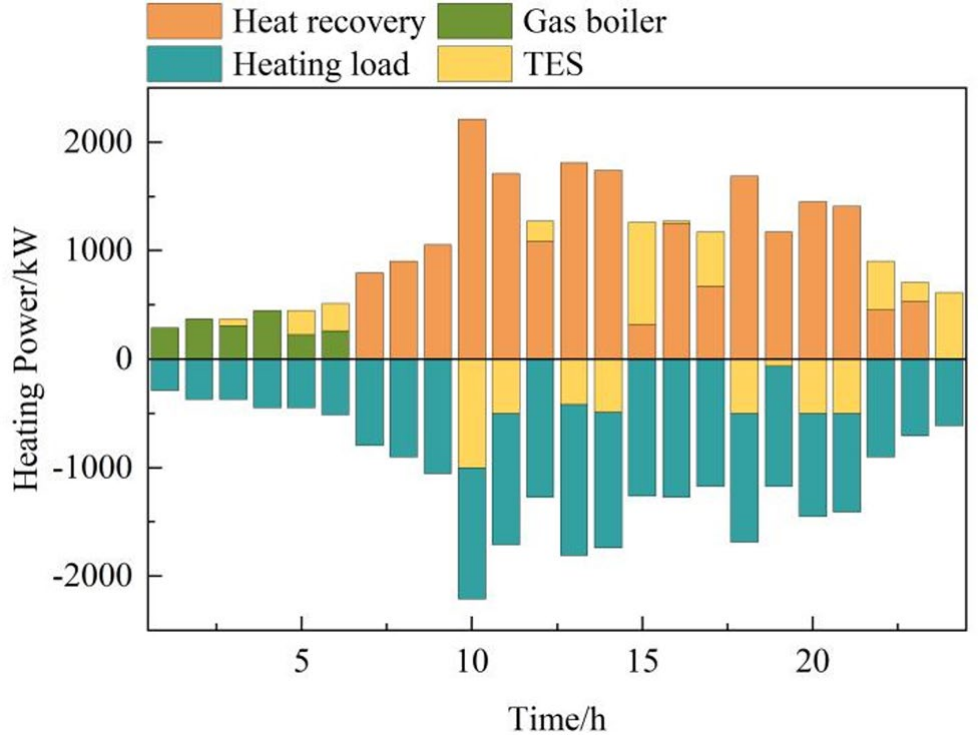
Thermal power optimization operation results in the day-ahead stage.

In peak and normal tariff periods, gas turbines produce power and waste heat, which is captured by recovery boilers to satisfy thermal loads, increasing efficiency. Thermal energy storage systems (TES) store excess heat and release it when needed, reducing reliance on gas-fired boilers. In off-peak periods, gas boilers and TES discharges meet thermal demands, as turbines operate less. Day-ahead thermal optimization results are in Fig. 7.

**Fig. 8:**
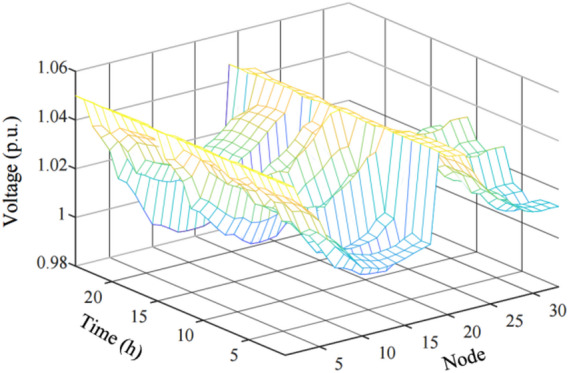
The voltage situation of each node in the day-ahead stage.

Nodes 11 and 24 serve as the interconnection points for CHP microgrids within the distribution network. Voltage at node 11 oscillates within the interval of [0.9880, 1.0309], and at node 24, it fluctuates within the range of [1.0202, 1.0337]. The voltage profiles across the nodes during the day-ahead optimization stage are illustrated in Fig. 8.

### Day-ahead stage optimization result analysis

Within the intraday horizon, a rolling optimization strategy is utilized to predict wind and solar power output with greater accuracy than the day-ahead stage, on an hourly basis, while accounting for imbalance penalties between the two stages. This method refines the day-ahead schedule by tweaking electricity procurement, gas turbine output, energy storage, and electric vehicle load response.

**Fig. 9:**
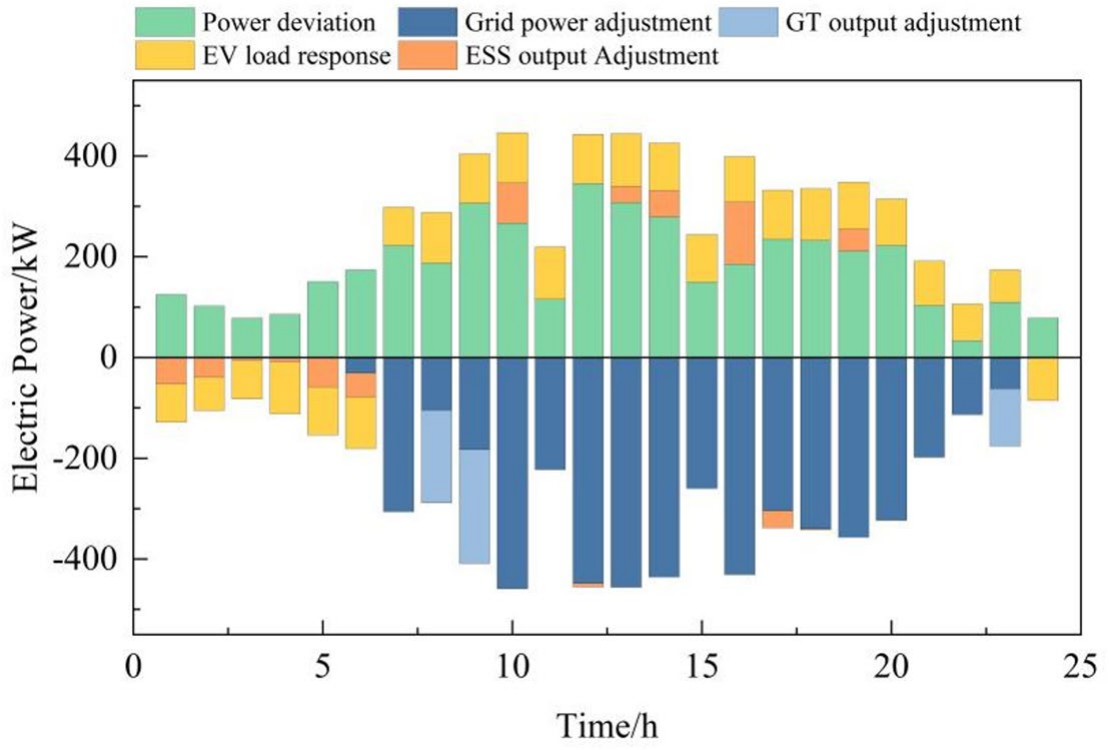
Electric power deviation adjustment.

The day-ahead stage’s conservative stance on wind and solar generation, due to robust optimization against uncertainty, is surpassed by the intraday stage’s increased renewable output. Electric vehicles and energy storage systems collaborate, employing V2G technology to absorb excess renewable energy and manage load during peak and off-peak pricing periods. This coordinated operation significantly reduces grid purchases and slightly adjusts gas turbine output, enhancing microgrid economic efficiency. The day-ahead power optimization results are presented in Figs. 9.

**Fig. 10:**
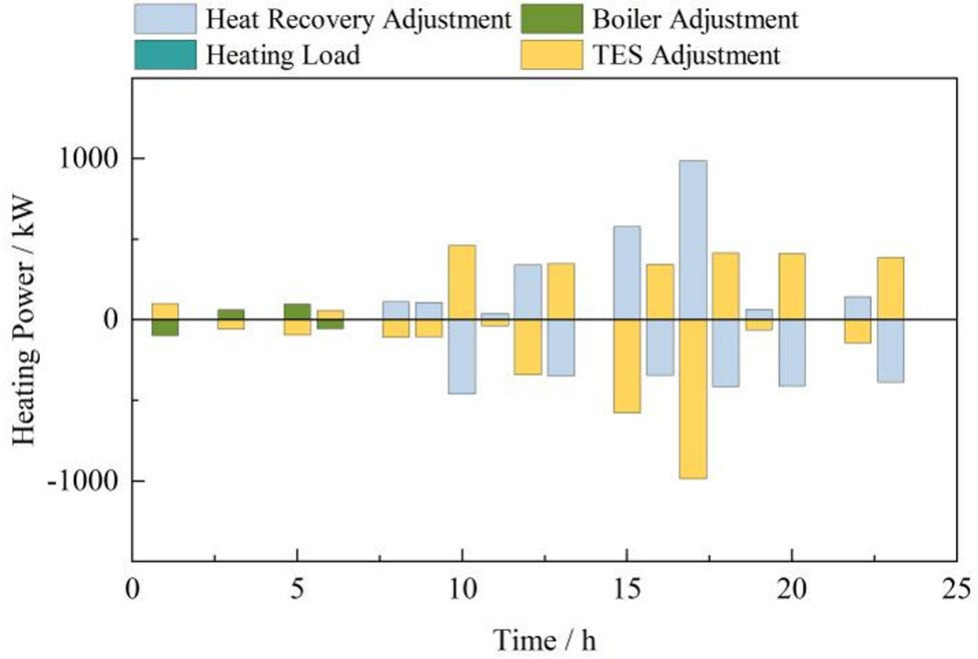
Thermal power deviation adjustment.

During off-peak tariff periods, thermal fluctuations are managed by thermal storage and gas boilers, with gas turbines inactive. In other periods, gas turbines operate continuously, and their waste heat is efficiently utilized by waste heat recovery boilers, which handle intraday thermal power adjustments along with thermal storage. Day-ahead thermal power optimization results are shown in Fig. 10.

**Fig. 11:**
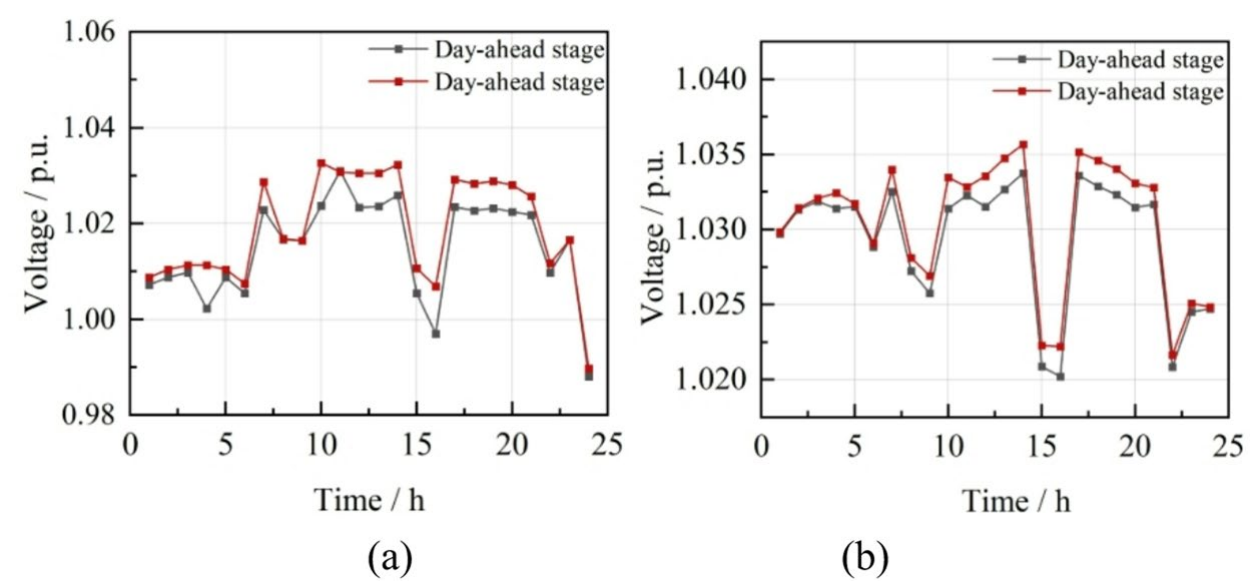
Voltage changes of node 11 and node 24 during the day. (**a**) Node 11, (**b**) Node 24.

Nodes 11 and 24, which connect CHP microgrids to the distribution network, have voltage fluctuations of 4.29% and 1.40%, respectively, similar to the day-ahead stage’s 4.29% and 1.36%. Despite robust optimization accounting for wind and solar uncertainty in the day-ahead stage, the intraday stage sees increased renewable output and reduced electricity purchases due to V2G response, leading to minimal voltage improvement in the microgrid, indicating a need for further optimization. Intraday voltage conditions at these nodes are detailed in Fig. 11.

### Real-time stage optimization result analysis

In the real-time adjustment stage, the certainty of wind and solar power output is further enhanced. This stage is based on 15-minute time-scale predictions of wind and solar power output. The energy conversion devices, such as gas turbines, waste heat recovery boilers, and gas boilers, determined in the intraday stage are substituted into the real-time optimization schedule. Real-time optimization of the microgrid is achieved by utilizing electricity purchases, electric energy storage device output, and incentive-based demand response guiding interruptible load quantities. The goal is to adjust and update the intraday optimization schedule to achieve optimal economic performance for the microgrid while ensuring voltage stability.

**Fig. 12:**
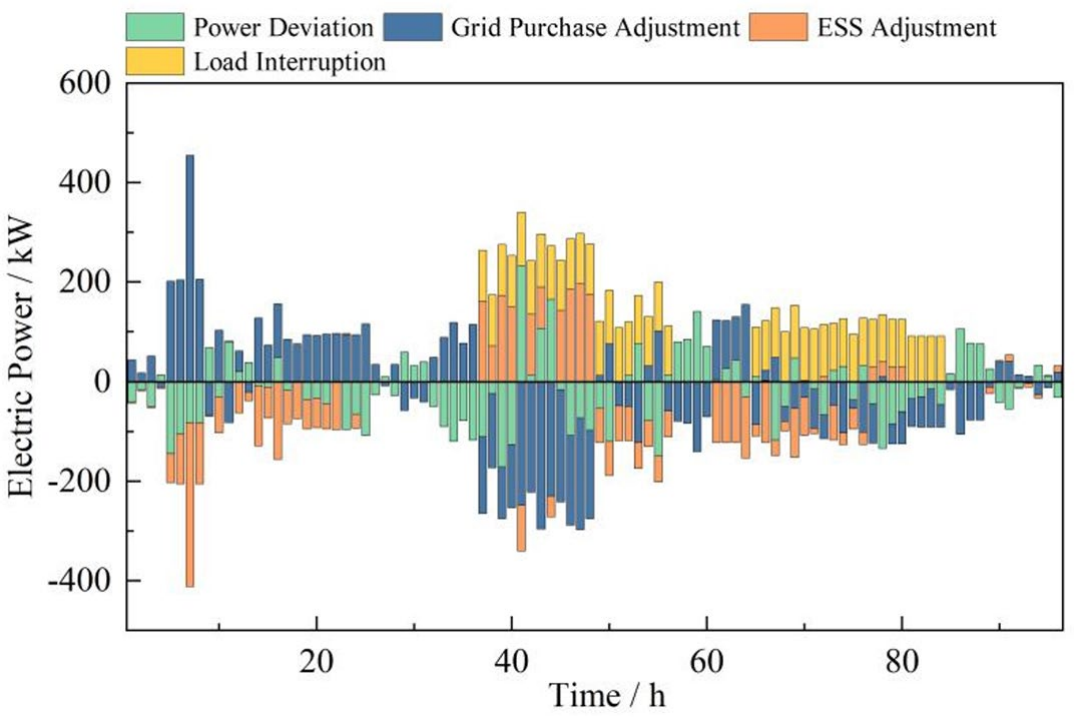
Electric power deviation adjustment in the real-time stage.

During periods of peak demand, the implementation of incentive-based demand response mechanisms is employed to manage interruptible loads that can rapidly respond. These loads, in coordination with the discharge of energy storage systems, fulfill a portion of the load requirements, thereby diminishing the reliance on grid-purchased electricity and enhancing the economic viability of the microgrid. In non-peak periods, to compensate for deviations in intraday and real-time wind and solar power generation, microgrid operators modulate electricity procurement and the charging/discharging cycles of energy storage systems to align with the generation from renewable sources, ensuring power balance within the grid. The real-time power deviation adjustments are detailed in Fig. 12.

**Fig. 13:**
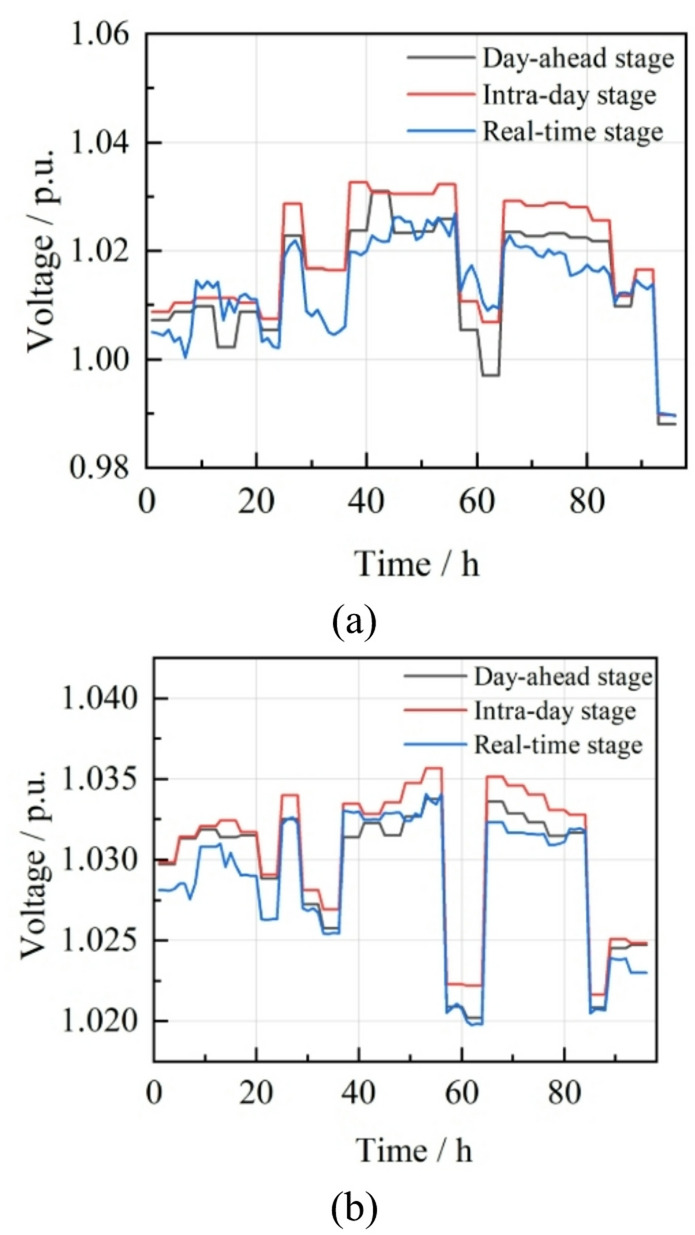
Voltage conditions of node 11 and node 24 in the real-time stage. (**a**) Node 11, (**b**) Node 24.

Nodes 11 and 24, which serve as the integration points for CHP into the distribution network, demonstrate voltage fluctuations within the ranges [0.9948, 1.0133] and [1.0098, 1.0190], respectively. The corresponding voltage fluctuation rates are 1.85% and 0.71%, indicating a marked enhancement in voltage stability relative to the day-ahead and intraday stages. The voltage profiles for nodes 11 and 24 during the real-time stage are presented in Fig. 13.

### ADN optimization operational state evaluation

This section aims to evaluate the operational status results of the ADN under different operational strategies through a comparative approach.

Solution 1: This solution considers only day-ahead optimization, utilizing the day-ahead scheduling plan.

Solution 2: This solution adopts a method that synergizes day-ahead optimization with intra-day optimization.

Solution 3: This solution incorporates a multi-time scale approach that integrates day-ahead optimization, intra-day optimization, and real-time optimization.

The optimized operational results of the ADN under the three solutions, along with relevant performance metrics, are presented in the Table 3.

**Table 3:** Optimization operation indicator data at different time scales.

Indicator	Details	Solution 1	Solution 2	Solution 3
Economic indicators	Purchased electricity cost (CYN)	43463.82	39900.13	37655.53
	Purchased gas cost (CYN)	19126.61	18771.95	18771.95
	Cost of line loss (CYN)	1222.85	1100.73	1452.52
	Demand response cost (CYN)	348.37	471.22	411.64
Economic indicators	Voltage fluctuation rate (%)	51.04	49.10	50.13
	Load fluctuation rate (%)	18.49	18.42	10.24
	Voltage fluctuation rate (%)	6.48	6.28	4.39

To rigorously validate the robustness and component-level benefits, three additional cases are simulated.

Case A: Implements day-ahead/intraday/real-time optimization without uncertainty sets.

Case B: Deactivates both price-based and incentive-based demand response.

Case C: Treats EVs as non-controllable loads (disables V2G).

The optimized operational results of the ADN under the above-mentioned solutions and cases, along with relevant performance metrics, are presented in the Table 4.

**Table 4:** Optimization operation indicator data in different methods.

Indicator	Solution 1	Solution 2	Solution 3	Case A	Case A	Case C
Total cost (CYN)	98,761	87,322	79,455	103,892	95,200	88,600
Renewable absorption (%)	82.1	88.5	93.2	76.4	85.8	82.5
Voltage fluctuation (%)	6.48	6.28	4.39	8.92	6.85	5.52

The deterministic model (Case A) incurs 30.7% higher costs than the proposed model (Solution 3) due to inadequate handling of renewable energy uncertainty, directly validating the necessity of the two-stage robust optimization framework in hedging against forecast errors. Removing demand response (Case B) results in a 19.8% increase in total costs compared to Solution 3, with renewable energy absorption dropping by 8.0% and voltage fluctuation worsening by 56.0%—confirming that demand response mechanisms play a critical role in load shaping and renewable energy accommodation. Disabling V2G (Case C) leads to a 11.5% higher total cost than Solution 3, with renewable absorption decreasing by 11.5% and voltage stability deteriorating by 25.7%. This demonstrates that EVs, when utilized as mobile energy storage through V2G, significantly enhance grid flexibility and renewable energy utilization.

In the context of Solution 1, where only day-ahead optimization is considered for operational performance metrics, a comprehensive analysis using set pair analysis can be conducted to derive the correlation degrees, as illustrated in expressions (72) and Table 5.

72 $$\left\{ \begin{gathered} {X_{\text{1}}}=[0.68698,0.83375,0.32383,0.16394] \hfill \\ {X_{\text{2}}}=[0.51041,0.18490,06481] \hfill \\ \end{gathered} \right.$$

**Table 5:** The correlation degrees of solution 1.

Eigenvalue	Metrics	Correlation degree				
		*z*_1_	*z*_2_	*z*_3_	*z*_4_	*z*_5_
*X*_1_	*x*_11_	0	0	0.2926	0.4675	0
	*x*_11_	0	0	0	0.6625	0.3375
	*x*_11_	0	0.5872	0.4128	0	0
	*x*_11_	0.3606	0.6394	0	0	0
*X*_2_	*x*_21_	0	0	0.7240	0.260	0
	*x*_22_	0.1510	0.8490	0	0	0
	*x*_23_	0	0	0.3798	0.3703	0

In the context of Solution 2, where both day-ahead and intraday optimization are considered for operational performance metrics, a comprehensive analysis using set pair analysis can be conducted to derive the correlation degrees, as illustrated in expressions (73) and Table 6.

73 $$\left\{ \begin{gathered} {X_{\text{1}}}=[0.65537,0.81819,0.29150,0.22175] \hfill \\ {X_{\text{2}}}=[0.49102,0.18418,0.62799] \hfill \\ \end{gathered} \right.$$

**Table 6:** The correlation degrees of solution 2.

Eigenvalue	Metrics	Correlation degree				
		*z*_1_	*z*_2_	*z*_3_	*z*_4_	*z*_5_
*X*_1_	*x*_11_	0	0	0.4116	0.3384	0
	*x*_11_	0	0	0	0.8181	0.1819
	*x*_11_	0.6950	0.6950	0.3050	0	0
	*x*_11_	0.9275	0.9275	0.0725	0	0
*X*_2_	*x*_21_	0.0299	0.0299	0.9701	0	0
	*x*_22_	0.8418	0.8418	0	0	0
	*x*_23_	0	0	0.4300	0.3200	0

In the context of Solution 3, where both day-ahead, intraday and real time optimization are considered for operational performance metrics, a comprehensive analysis using set pair analysis can be conducted to derive the correlation degrees, as illustrated in expressions (74) and Table 7.

74 $$\left\{ \begin{gathered} {X_{\text{1}}}=[0.65537,0.81819,0.29150,0.22175] \hfill \\ {X_{\text{2}}}=[0.49102,0.18418,0.62799] \hfill \\ \end{gathered} \right.$$

**Table 7:** The correlation degrees of solution 3.

Eigenvalue	Metrics	Correlation degree				
		*z*_1_	*z*_2_	*z*_3_	*z*_4_	*z*_5_
*X*_1_	*x*_11_	0	0	0.4064	0.3436	0
	*x*_11_	0	0	0	0.8181	0.1819
	*x*_11_	0	0.3845	0.6155	0	0
	*x*_11_	0.0629	0.9371	0	0	0
*X*_2_	*x*_21_	0	0	0.7469	0.0031	0
	*x*_22_	0.9763	0.0237	0	0	0
	*x*_23_	0	0.2022	0.7978	0	0

Upon obtaining the correlation degrees of the status levels for feature indicators across various solutions, expression (53) is utilized to perform a weight transformation. Subsequently, these transformed weights are integrated with the correlation results derived from the aforementioned set pair analysis to ascertain the correlation degrees of the optimized operational state variables *X*_1_ and *X*_2_ for the ADN under different solutions.

Feature quantity correlation results in different solutions are shown in Table 8.

By employing Eqs. (67) and (68), the correlation calculation results of the ADN’s optimized operational state feature indicators across different time scales are integrated to derive the basic probability assignment calculation outcomes. The correlation results for the operational states at various time scales are respectively presented in Eqs. (75), (76), and (77).

**Table 8:** Feature quantity correlation results.

Solution	Eigenvalue	*z*_1_	*z*_2_	*z*_3_	*z*_4_	*z*_5_	$m\left( \theta \right)$
Solution 1	*X*_1_	0.0741	0.2504	0.1498	0.2801	0.0869	0.100
	*X*_2_	0.0401	0.2254	0.1990	0.0989	0	0.3374
Solution 2	*X*_1_	0	0.3371	0.1711	0.2878	0.0467	0.1000
	*X*_2_	0.0423	0.2291	0.2453	0.0819	0	0.3374
Solution 3	*X*_1_	0.0131	0.2756	0.2211	0.2869	0.0464	0.1000
	*X*_2_	0.2653	0.0556	0.3043	0	0	0.3374

75 $${U_{{\text{DA}}}}=[0.0652,0.3330,0.2042,0.2691,0.0597,0.0687]$$

76 $${U_{{\text{IA}}}}=[0.0081,0.4180,0.2386,0.2474,0.0303,0.0648]$$

77 $${U_{{\text{AT}}}}=[0.0737,0.2439,0.3690,0.2076,0.0335,0.0722]$$

Based on the derived Eqs. (75), (76), and (77), the numerical values within these equations represent the membership degrees corresponding to the five defined status levels, with the final value indicating the degree of uncertainty. By applying Eq. (71), the membership degrees associated with different status levels are equivalently replaced with fractional values. The numerical values are negatively correlated with the optimized operational status of the ADN, meaning that a lower status index indicates a better optimized operational status.

Combining the results, the ADN’s optimized operational status index for day-ahead optimization only is 38.27, for day-ahead and intra-day optimization is 37.91, and for day-ahead, intra-day, and real-time optimization is 36.53. These status indices, along with the defined status levels, demonstrate that the ADN’s optimized operational status across the three different time scales is relatively optimal. When considering both economic and reliability factors, Solution 3 is superior to Solution 2, and Solution 2 is superior to Solution 1, further validating the feasibility of multi-stage optimization for the ADN.

## Conclusion

This paper presents a robust and evidence-based approach to the optimization and evaluation of ADN that incorporate EVs and CHP systems. The multi-stage operation optimization model and the evaluation methodology developed in this study have demonstrated significant improvements in the economic efficiency and operational reliability of microgrids. Below are the specific conclusions derived from this research:

1. Optimization Model Effectiveness: The multi-stage operation optimization model, which includes day-ahead scheduling, intraday correction, and real-time adjustment stages, has been proven effective in reducing the impact of renewable energy output prediction errors on ADN scheduling. This model facilitates the integration of renewable energy sources and enhances the overall efficiency of ADN dispatch.
2. Economic and Reliability Enhancement: Simulation results on the IEEE 33-node distribution system have shown that the multi-stage operation optimization model can significantly improve the economic performance and reliability of ADN operations. This indicates the model’s potential to provide substantial benefits in practical ADN management.
3. Evaluation Model Validation: The evidence-based evaluation model, which combines set pair analysis and evidence theory, has been successfully applied to assess the multi-stage operation status of the ADN. The evaluation results, quantified by a status index, have validated the superiority of a multi-stage operation approach over other strategies in terms of both economic and stability aspects.

Future research endeavors could concentrate on integrating cutting-edge energy storage solutions, which are pivotal for bolstering the adaptability and dependability of ADN. Additionally, the exploration of artificial intelligence and machine learning methodologies, including deep learning and reinforcement learning, could be pursued to construct more advanced forecasting models.

## Supplementary Information

Below is the link to the electronic supplementary material.

Supplementary Material 1

## Data Availability

Data is available on request. To request data, contact the corresponding author Yunshou Mao, E-mail: maoyunshou@163.com.
