# Supplementary material for: Robust modeling and evidence-based evaluation method for a active distribution network with EVs and CHPs
Source: Sci Rep. 2025 Nov 21;15:41221. doi: 10.1038/s41598-025-25084-3 (PMC12638864; doi:10.1038/s41598-025-25084-3)
Supplement: Supplementary file 1 — Supplementary Material 1 [file 41598_2025_25084_MOESM1_ESM.doc]

**APPENDIX A**

**
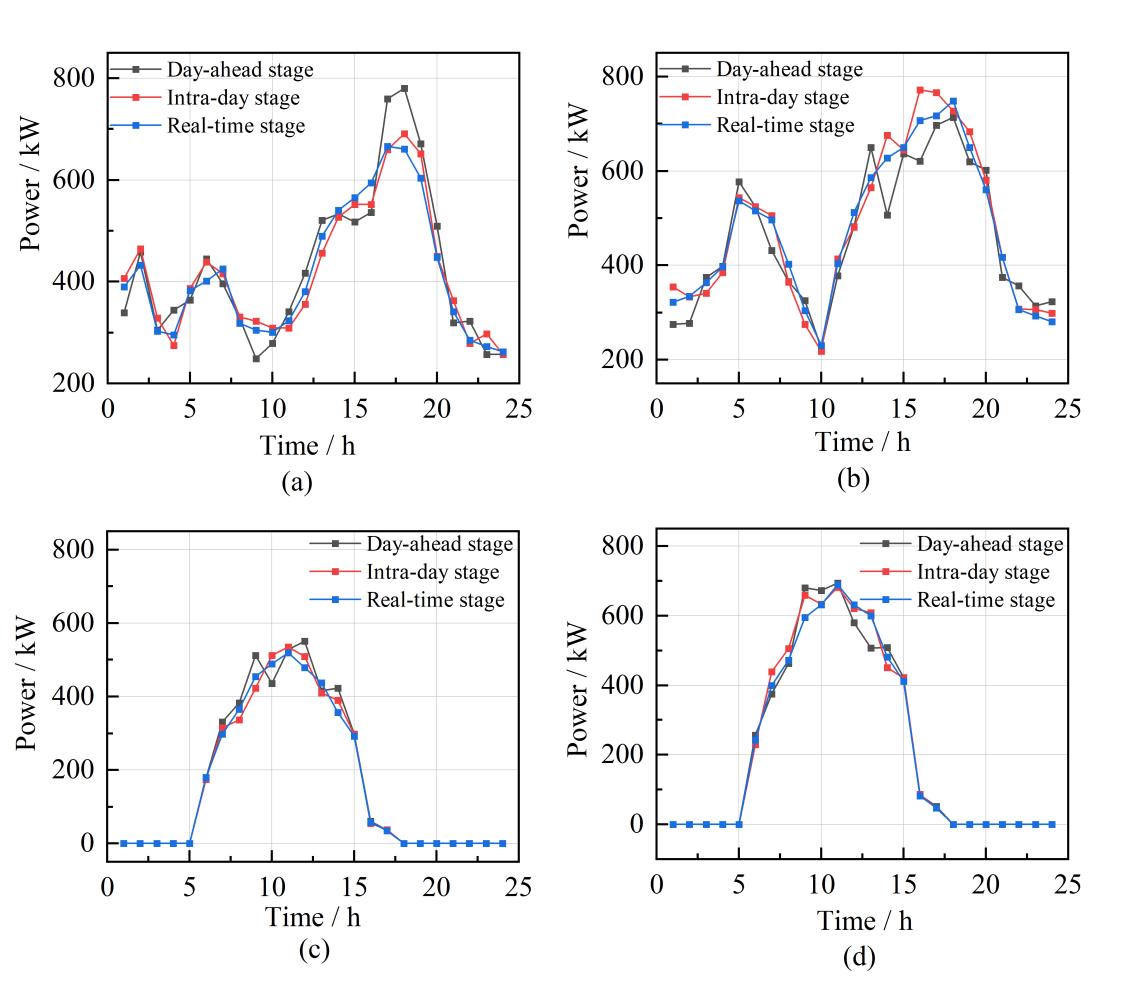

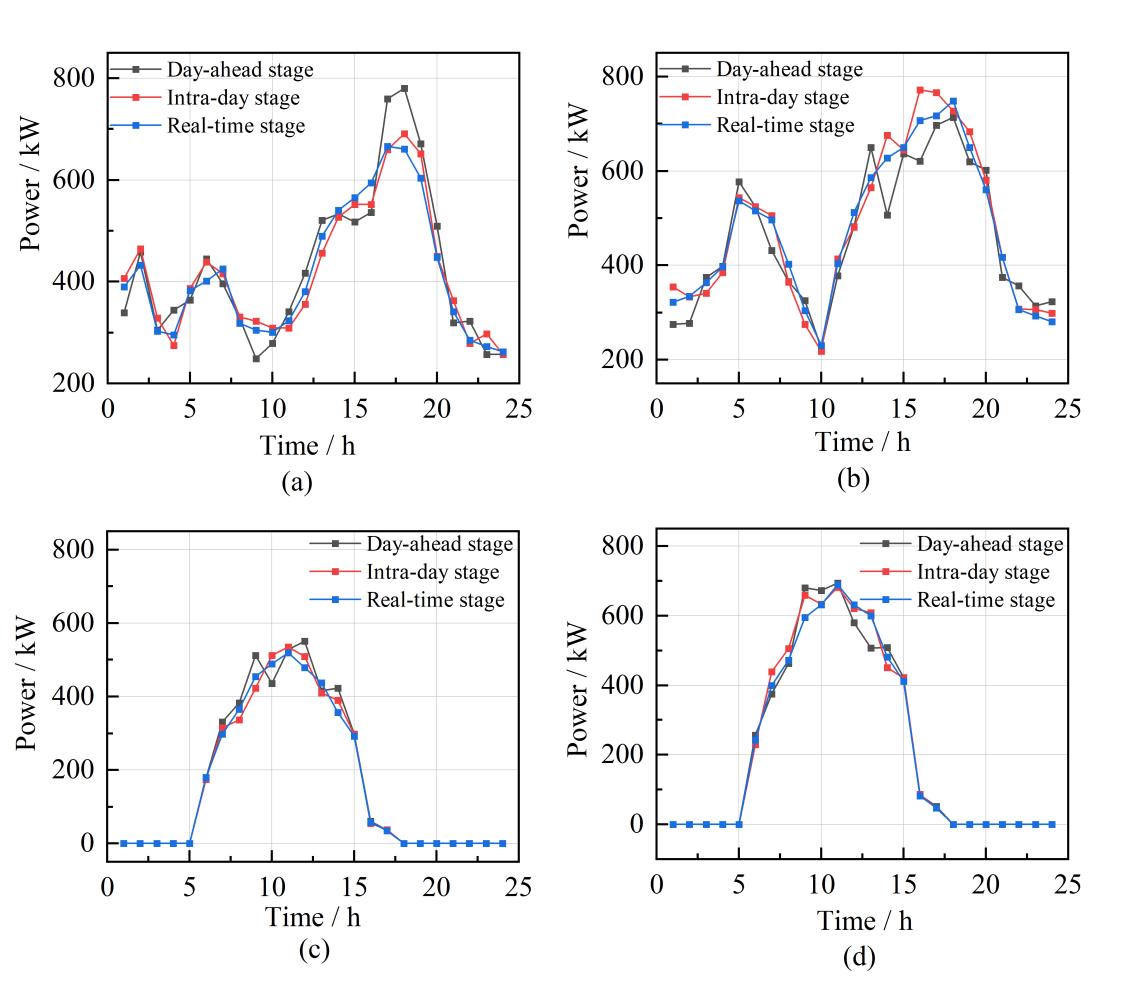
**

**
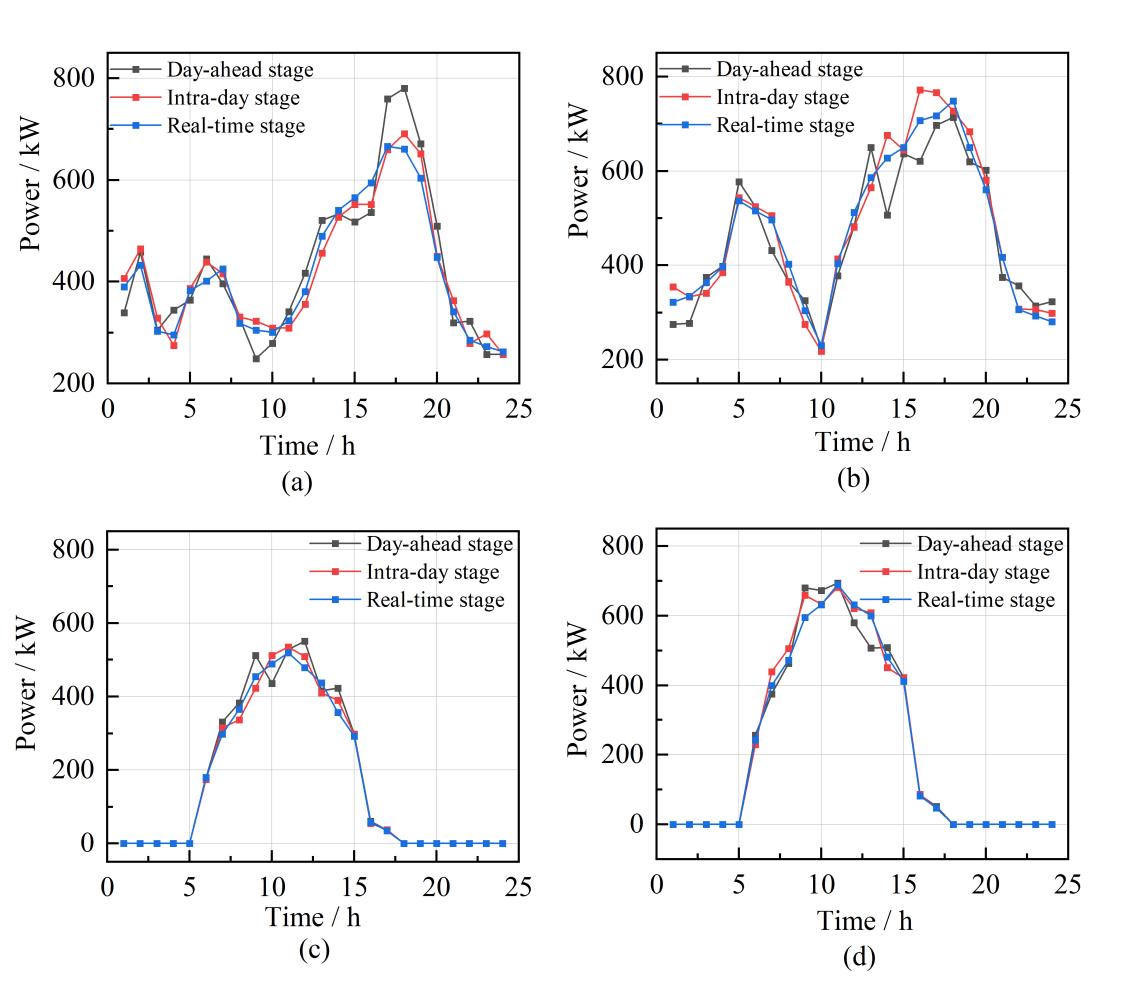

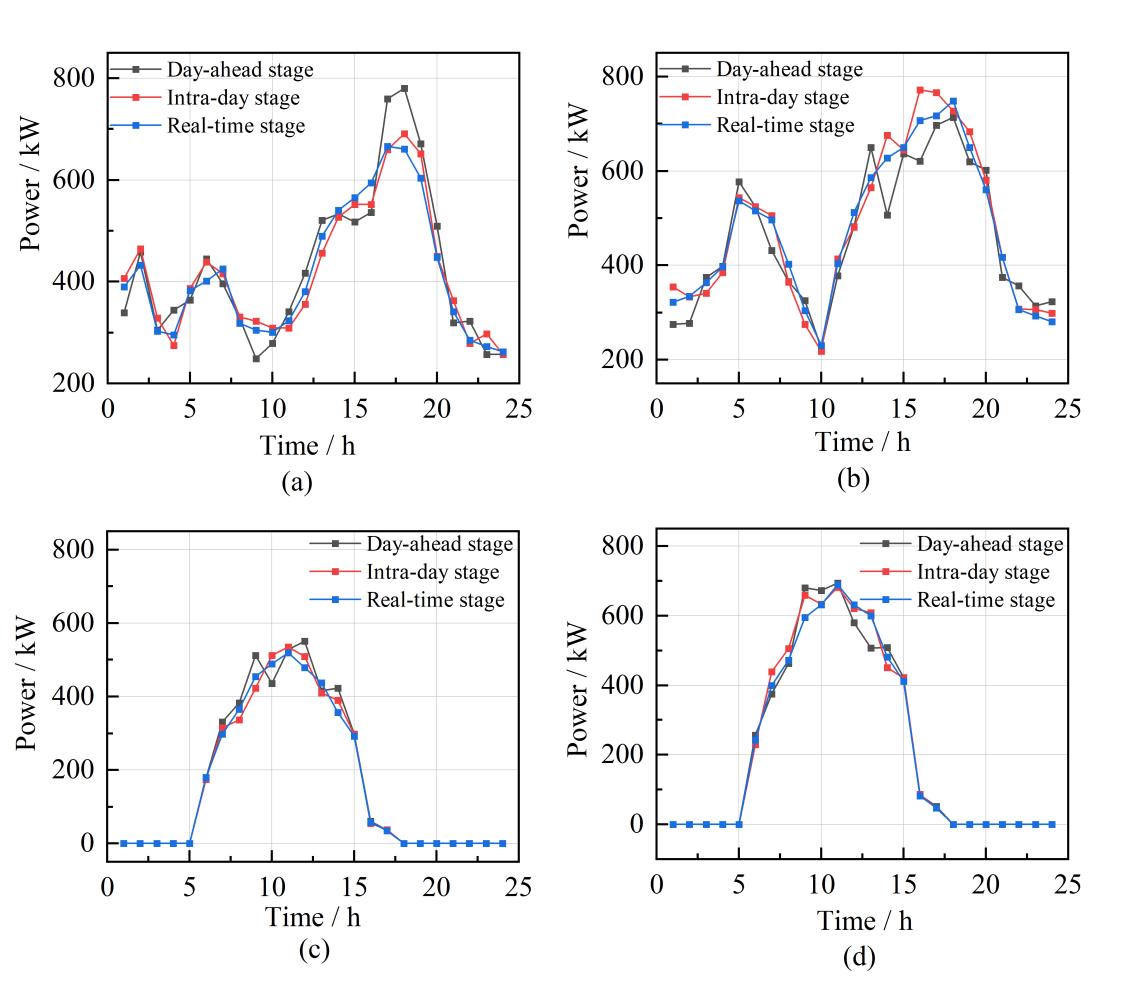
**

Figure A1 WT output and PV output forecast in ADN in different time stage: (a) WT power output in node 15; (B) WT power output in node 22; (c) PV power output in node 8;(d) PV power output in node 31

**
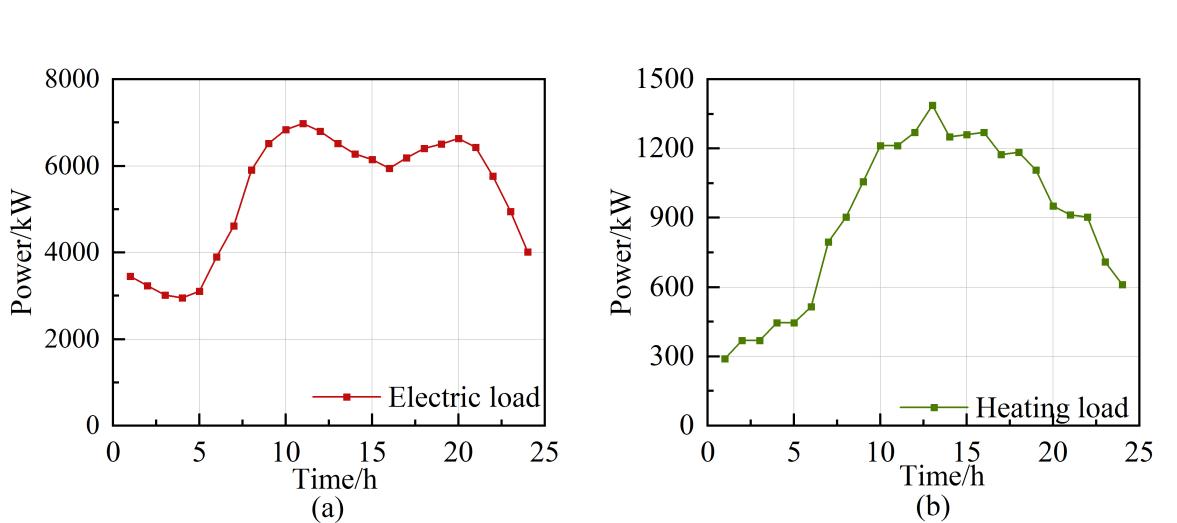
**

**
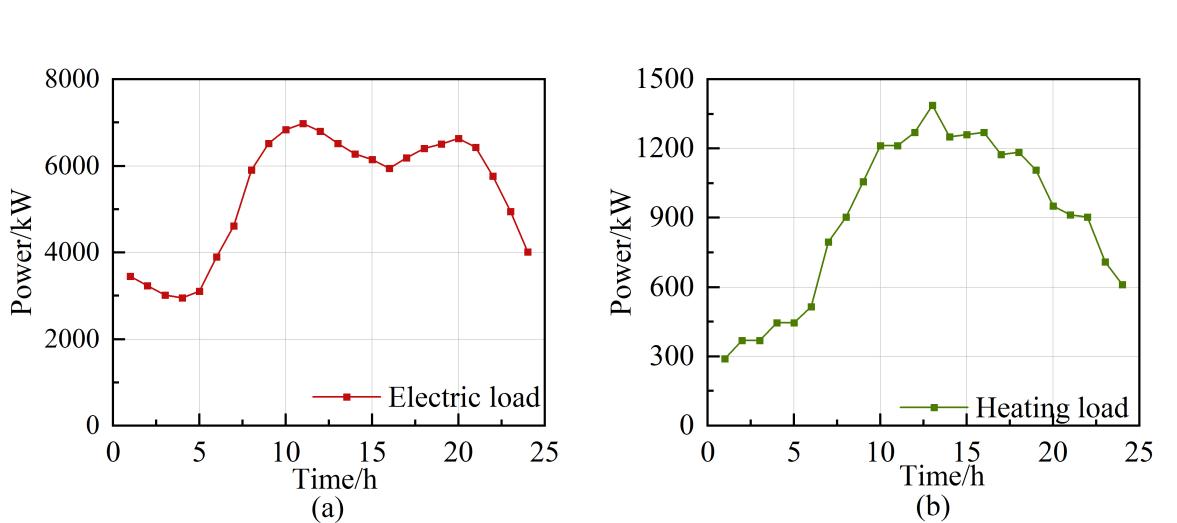
**

Figure A2 Load forecast of ADN: (a) Electric load; (B) Heating load.

**Table A1. Combined heat and power mircogrid equipment and parameters**

| Unit | Parameter | Value |
| --- | --- | --- |
| Gas turbine | Rated power (kW) | 2000 |
|  | Power generation efficiency | 0.35 |
| Electric energy system | Rate capacity (kW·h) | 2000 |
|  | Initial energy (kW·h) | 600 |
|  | Minimum energy (kW·h) | 300 |
|  | Maximum energy (kW·h) | 1800 |
|  | Self-discharge rate | 0.001 |
|  | Maximum charging power (kW) | 550 |
|  | Minimum discharging power(kW) | 550 |
|  | Charging/discharging efficiency | 0.95 |
| Heat recovery system | Rated power(kW) | 2400 |
|  | Thermal recovery efficiency | 0.7 |
| Gas boiler | Rated power(kW) | 1500 |
|  | Thermal efficiency | 0.85 |
| Thermal energy system | Rated capacity(kW·h) | 2500 |
|  | Initial energy (kW·h) | 750 |
|  | Minimum energy (kW·h) | 375 |
|  | Maximum energy (kW·h) | 2250 |
|  | Self-discharge rate | 0.001 |
|  | Maximum charging heat power(kW) | 550 |
|  | Minimum charging heat power(kW) | 550 |
|  | Charging efficiency | 0.95 |
